# New insights for understanding spatial patterning and formation processes of the Neanderthal occupation in the Amalda I cave (Gipuzkoa, Spain)

**DOI:** 10.1038/s41598-020-65364-8

**Published:** 2020-05-26

**Authors:** Laura Sánchez-Romero, Alfonso Benito-Calvo, Ana B. Marín-Arroyo, Lucía Agudo-Pérez, Theodoros Karampaglidis, Joseba Rios-Garaizar

**Affiliations:** 1 Human Evolution Research Center, 3101 Valley Life Sciences Building, University of California, Berkeley, CA 94720 USA; 2 Centro Nacional de Investigación sobre la Evolución Humana, P° Sierra de Atapuerca, 3, 09002 Burgos, Spain; 3 EVOADAPTA Group, Instituto Internacional de Investigaciones Prehistóricas de Cantabria, Universidad de Cantabria (UC), Av. Los Castros, 52, Santander, Spain

**Keywords:** Archaeology, Archaeology

## Abstract

The Level VII of Amalda I cave (Gipuzkoa, Spain) represents one of the latest Middle Palaeolithic occupations in the Cantabrian Region. It is characterized by the presence of Middle Palaeolithic lithic industry and animal remains, with clear evidences of anthropic and carnivore manipulation. At this site, the Neanderthal presence has been questioned in relation to the role of carnivores in the accumulation of large, medium-sized and small mammals. It has also been proposed that the Neanderthal occupation could have consisted of short-term occupations, where different activities took place in a structured space within the cave. However, all hypotheses lacked any integrative analysis of the site formation processes. With the aim of understanding these processes, a combination of spatial techniques, based on GIS and inferential statistics (density analysis, hotspots tools and palaeotopographic reconstruction), along with the taphonomic study of identifiable and non-identifiable macromammals remains, were employed. This study has revealed distinct use of the cave space by Neanderthals and carnivores. The major concentrations of lithics and medium-size mammal remains were clearly accumulated by humans at the cave entrance, while the small-size mammals were gathered by carnivores in an inner zone. The activities of the Neanderthals seem to be distinctly structured, suggesting a parallel exploitation of resources.

## Introduction

The Neanderthal presence in the Cantabrian region has been documented from the end of the Middle Pleistocene to the MIS4 and MIS3, as we can see in sites such as Lezetxiki^1,2^, Arlanpe^3^, Axlor^4–7^, Aranbaltza^8^, El Castillo^9,10^, El Esquilleu^11–14^, Cueva Morín^15^, Covalejos^16^-10), El Sidrón^17^ or La Viña^18^. The data recovered from all these sites indicate a great variability in lithic technology, subsistence strategies and landscape uses, suggesting that Neanderthals successfully adapted to different climatic and environmental contexts, experiencing also important cultural changes, evidencing the complexity and particular history of these groups.

Amalda I is a cave with a long sequence, with a single Middle Palaeolithic level (Level VII) with undisputed evidence of human and carnivore activity, both as bone accumulators. This has given rise to a certain amount of debate about the nature of the occupation and the relevance of the carnivores in the taphocenosis of the deposit^19–21^. Previous analysis of the lithic assemblage and its spatial distribution has interpreted this deposit as an occasional logistic occupation for the exploitation of local resources, or as a temporary refuge for Neanderthal groups moving between regions^22,23^. This earlier study did not include a detailed analysis of the site formation processes and the role of the carnivores, not only as *bone accumulators* but also in the possible postdepositional alterations after human occupation^24^. The Amalda I site has great potential for assessing the spatial organisation of the activities and the space use by Neanderthals, which has essential implications for understanding their social organisation, such as the existence of well-structured productive processes, the division of labour or the existence of some kind of principles in the organisation of the habitat. The present work aims to identify the spatial organisation, occupation pattern and factors that could have acted in the formation of the assemblage, as well as the alteration processes afterwards. This enables us to observe that the human and carnivore activities can be spatially distinguished, and that the human activity seems to be structured, both by the location of their main accumulations and the type of activity carried out.

## Context

**Figure 1:**
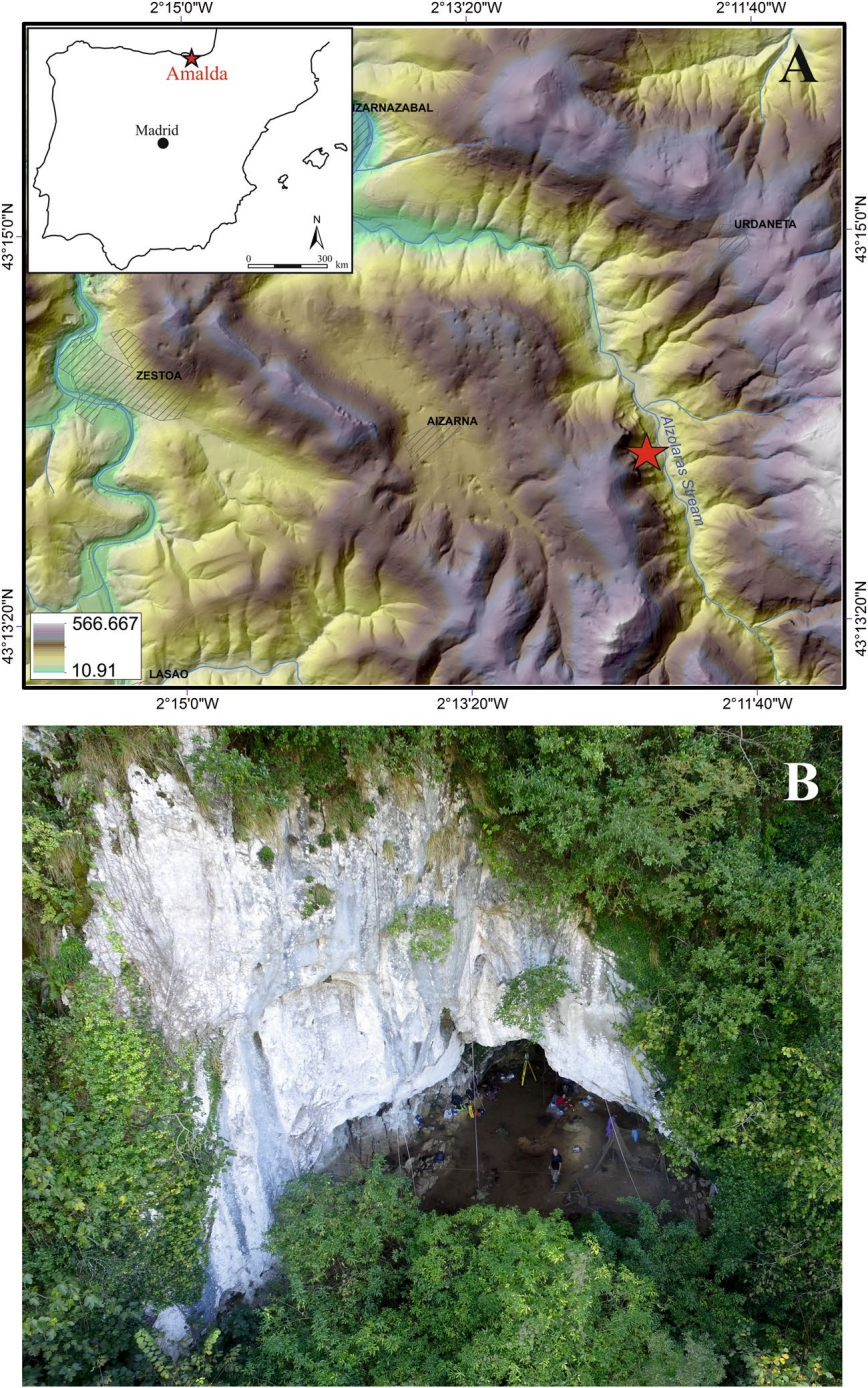
(**A**) Digital elevation model (DEM) showing the position of Amalda I site in the context of the Alzolaras valley (Gipuzkoa, Basque Country). (**B**) Photo of the cave entrance taken with drone.

The Amalda I cave (Zestoa, Gipuzkoa) is located on the western hillside of the Alzolaras valley (Fig. 1), 110 m from the current base level of the Alzolaras stream, in a closed subsidiary valley of the Urola River, 11 km away from the current coastline. The Alzolaras gorge is deeper and narrower upstream from the cave, becoming wider towards the position of the site^19^. The Alzolaras stream runs 4 km until its confluence with the Urola River, which in turn currently flows towards Zumaia and into the Cantabrian sea. This connexion with the Urola River could have served as a natural communication route with the Aizkorri mountain range that allows the access to the Ebro basin and Llanada Alavesa. The Urola River could also have served as access route to the coast^23^. Amalda I opens perpendicularly onto the river in a steep front of Urgonian limestones, and its formation is a consequence of the dissolution of these limestones to leave a subvertical joint (fracture) (W-E direction)^19–23^. The cave has a characteristic triangular entrance, which gives way to a wide gallery 50 m long that narrows further in. The description of the lithostratigraphic sequence of Amalda I is based on the observations and analysis conducted during the excavation fieldwork developed between 1979 and 1984^19^ and the reworking of one section by ourselves in 2017 (Supplementary, Fig. S1). Level VII is characterized by a silty-clay matrix, with a low percentage of sand, no cementation and some angular gravels of limestone at the top, with Middle Palaeolithic lithic industry and faunal remains (Supplementary, Fig. S1).

The Level has been recently dated between ca. 44,500 and 42,600 uncal BP by 14C AMS of ultrafiltered collagen samples^25^, being one of the most recent presences of Middle Palaeolithic in the region. From the same Level, another dated sample gave a result of 28,640 ± 310 (OxA-32425) (Table 1), which jars with the Middle Palaeolithic dating but is very close to the results obtained for the immediately superior Gravettian Level VI^25^. A limited admixture between Levels VI and VII, also detected in the lithic assemblage^23^, would explain this odd date.

**Table 1:** 14C AMS (ultrafiltered) results of the bone samples from the Level VII of Amalda I^25^.

Sample	Species	Bone	Modification	Lab Code	Date (BP)	Error	%C	%N	C:N	d13C
A.12D.124	*Equu*s sp.	Metacarpal	Impact notch	OxA-32425	28640	310	42.4	4.1	3.2	−20.8
A8G.204.13	*Cervus elaphus*	Metatarsal	Anthropogenic breakage	OxA-32500	44500	2100	42.1	3.8	3.4	−20.2
7 G.221.16.X62.Y82	*Cervus elaphus*	Tibia	Anthropogenic breakage	OxA-34933	42600	1600	43.7	15.9	3.2	−21.2

## Results

### Dating

Three carnivore-modified bones were selected for dating, two from Altuna’s collection and one yielded from the excavation in 2017 by our team. The samples from the former come from the central part of the excavated area (9C and 6D) and were selected from the top and bottom of Level VII. These remains were identified as *Rupicapra rupicapra* and had clear gnawing marks (Table 2). The sample from the 2017 fieldwork comes from a marginal area of the excavation (G11-12). These remains were also identified as *Rupicapra rupicapra* and all of them had gnawing and digestion traces (Table 2). Two of the three dates (Beta-451923 and Beta-451419) are consistent with the outlier obtained in 2018 (OxA-32425) (Table 1)^25^, which might suggest that the carnivore activity was coeval to the Early Gravettian occupation of the site. The third date (Beta-451922) seems to indicate that carnivores roamed the cave when it was unoccupied by humans, between the Late Middle Palaeolithic and the Early Gravettian (Table 2). These results also indicate that some admixture exists between Middle Palaeolithic and Gravettian materials.

**Table 2:** 14C AMS (ultrafiltered) results of the dated carnivore modified bone samples from the Level VII of Amalda I.

Sample	Species	Bone	Modification	Lab Code	Date (BP)	Error	%C	%N	C:N	d13C
A.9C.171	*Rupicapra rupicapra*	Tibia	Gnawed	BETA-451923	28720	140	37.26	13.43	3.2	−20
A.6D.208	*Rupicapra rupicapra*	Falanx	Gnawed	BETA-451922	35590	270	39.9	14.47	3.2	−19.8
A.17.10020	Indetermined	Fragment	Digested	BETA-481419	28240	150	41.74	14.9	3.3	−20

### Taphonomic analysis

**Figure 2:**
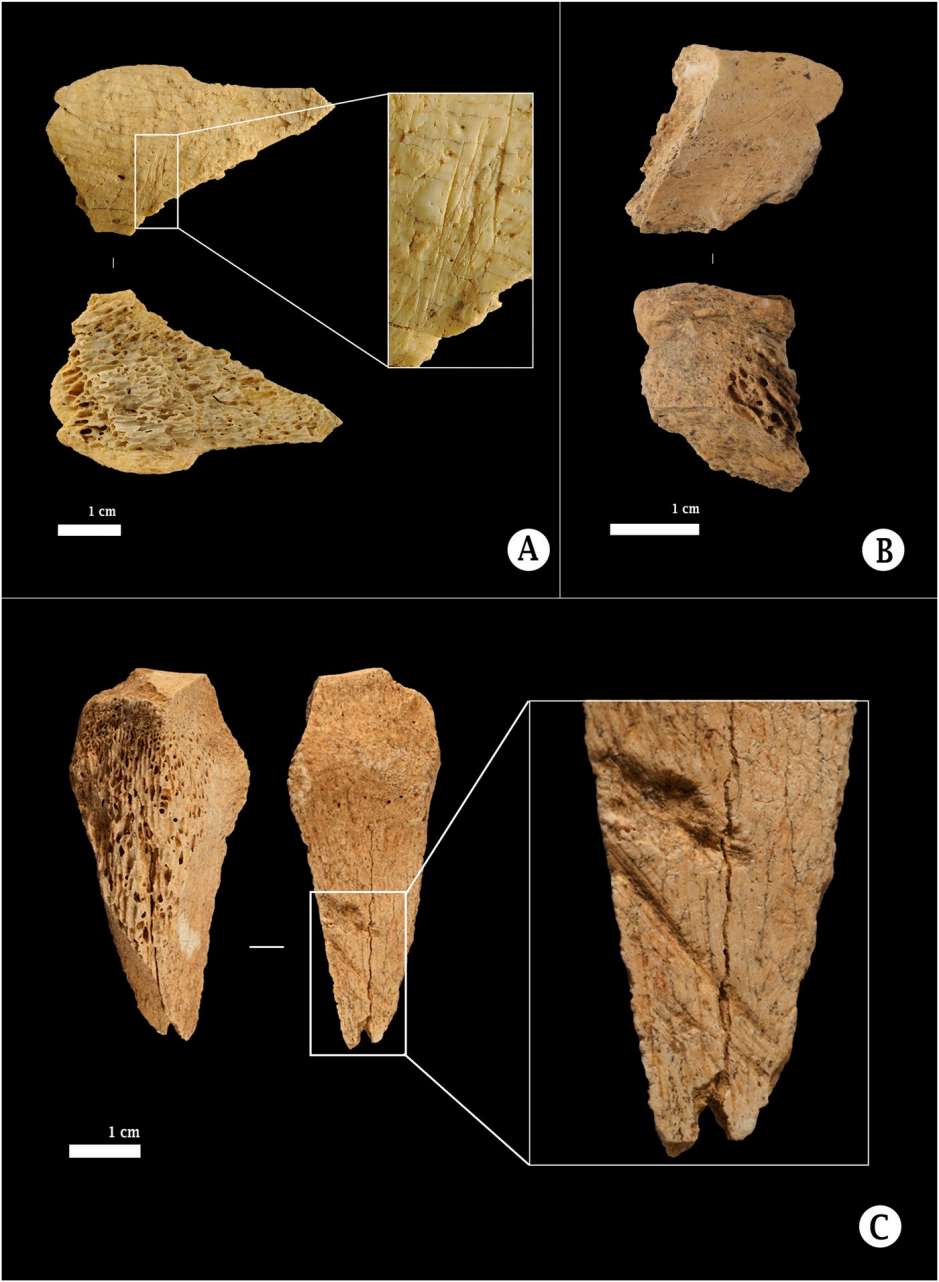
Anthropogenic modifications identified within the non-identifiable faunal assemblage of Level VII at Amalda I. (**A**) Metaphysis of large-size mammal with cut marks. (**B**) Metapodial of *Cervus elaphus* with anthropic fresh breakage and cut marks (**C**) Proximal radius of *Equus* sp. with oblique cut marks in the cranial side. Image: Lucía Agudo.

The results obtained refer to the non-identifiable elements of the assemblage, a total number of 4,589 fragments, including antler, bones and teeth. In terms of taxonomy, despite the fragmentation (the average length is 0.6 cm), it was still possible to attribute 18% of the assemblage to mammal-size categories and some specific ungulates (82% was completely indeterminate). Only 2% were identified as ungulates, such as *Bos/Bison* sp., *Equus* sp., *Cervus elaphus*, *Capra pyrenaica* and *Rupicapra rupicapra*. Also, some carnivores were identified, such as *Ursus* sp., *Canis lupus* and *Vulpes vulpes*. Apart from those, some avifauna and mustelids were also identified. Due the preservation state, 16% of the assemblage was only categorised as large, medium, small and very small mammals that included both ungulates and carnivores (Table 3). Regarding the taphonomy of the non-identifiable faunal elements, 10% showed anthropogenic modifications, including percussion marks on long-bone shafts (0.2%), fresh breakage patterns (1.2%), flakes (2.3%), cut marks (1.3%) and thermoalterations (7.4%) (Fig. 2, Table 4). On the other hand, carnivore traces on bones were better represented, since 3% of them showed carnivores marks, including scores, furrowing and punctures, 24% of them digestion traces and only 0.4% show both marks and digestive traces (Table 4). This high number of tiny digested bones can only be the result of defecation onto the scats on-site by the carnivores responsible for consuming the animal carcasses.

**Table 3:** Taxonomy of the non-identifiable faunal assemblage in terms of Number of Remains (NR) and their percentage of representation.

	NR	%
Ungulates	73	1.59
Carnivores	10	0.22
Avifauna	12	0.26
Mustelids	2	0.04
Large size-mammals	194	4.23
Medium size-mammals	204	4.45
Small size-mammals	298	6.49
Very small size-mammals	38	0.83
Indeterminate	3758	81.89
TOTAL	4589	100

**Table 4:** Anthropogenic and carnivore modifications identified within the non-identifiable faunal assemblage in terms of Number of Remains (NR) and their percentage.

	N	%
**Anthropogenic modifications**		
Fresh breakage	57	1.2
Bone flakes	101	2.2
Cut marks	8	0.2
Thermoalterations	340	7.4
Both cut marks + carnivore marks	12	0.3
**Carnivore modifications**		
Carnivore marks (scores, punctures, furrowing)	116	2.5
Digestive traces	1122	24.4
Both carnivore marks + digestion	18	0.4

### Palaeotopographic reconstruction of Level VII

**Figure 3:**
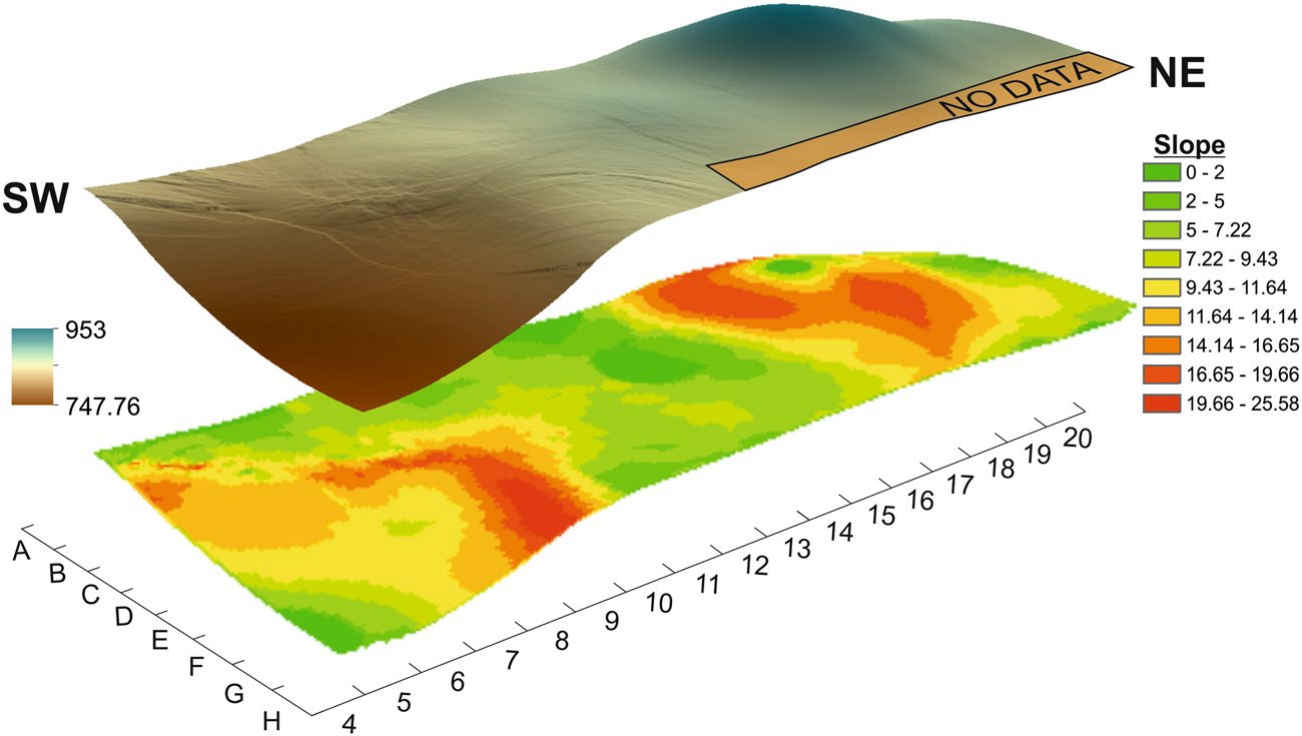
Palaeotopographic reconstruction and slope map of the Level VII from Amalda I.

The reconstruction of the palaeotopography of the Level VII was based on 595 points located at the base of the Level VII. The results obtained for this level show a continuous surface characterised by a mean slope of 9.54°, where it is possible to observe a higher part located to the NW, towards to the interior of the cave, and a lower zone located to the SE, at the entrance (Fig. 3).

### Distribution patterns

**Figure 4:**
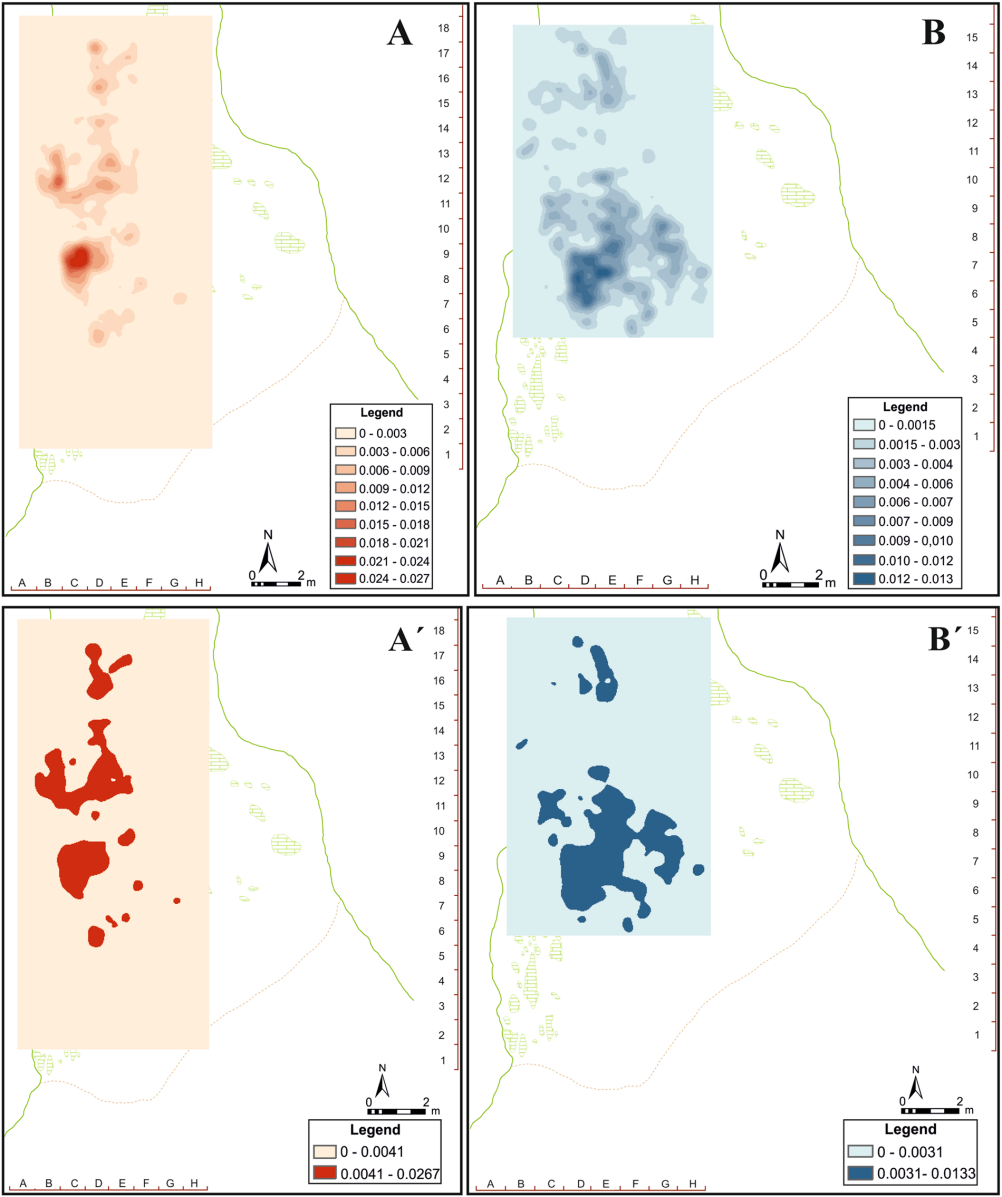
Kernel density analysis and Jenks classification method applied to faunal (**A**, A’) and lithic (**B**, B’) remains from the Level VII, Amalda I cave.

The application of kernel density analysis indicates that the zone with the maximum concentration of lithic remains does not coincide with the zone with maximum concentration of faunal remains. The two clusters are located facing each other and separated by a gap (Fig. 4), in which there is a small amount of material, delimited by the two main concentrations of lithic and faunal remains. The clustered nature of the data was verified through the application of several statistical tests according to the number of points (n = 1972) per square (n = 96). Proceeding in this way, the chi-squared (X^2^) and Kolmogorov-Smirnov (K-S) tests revealed that both lithic and faunal remains are grouped (or clustered), showing lower critical values than the significance level (*p* = 0.95) (Supplementary, Section 2). Additionally, other analyses were carried out to delve into the distribution of the remains, due to the apparently differentiated patterns observed between faunal and lithic industry, as well as the clustered pattern resulting from the different statistical tests applied. Thus, we have analysed the Average Nearest Neighbour (ANN) of the projected points, as well as the Global Moran’s I, incremental spatial autocorrelation and the Ripley’s K function according to the distribution of points and squares (Supplementary, Table S1).

### Definition of the main clusters

The identification and definition of the main accumulations of materials were made possible through hotspots analysis, calculating the Getis-Ord Gi* and the Anselin Local Moran’s I statistics according to the variable of maximum length. These techniques provided the statistical significance of the clusters that comprise high (*hot*) and/or low (*cold*) values as a function of the value of the maximum length and their spatial location (Supplementary, Figs. S2 and S3).

### Characterization of the groups

The clusters related to the lithic industry have been named CL1 and CL2, while the clusters of faunal remains are CF1 and CF2. Regarding the lithic clusters, there are some differences between them, such as the number of remains making up each. Although both are mainly composed of small remains, the minimal length being equal or less than 10 mm (Table 5). The most abundant raw material in Level VII is flint, although there are many others such as argillite, quartzite, quartz, limonite and mudstone. Thus, in both clusters the most represented raw material is flint. Regarding the support, fragments dominate the assemblage of both clusters, followed by flakes and resharpening flakes (Supplementary, Table S4). With regard to the types of tools, the non-retouched pieces clearly dominate the record, both in CL1 and CL2. Side-scrapers are the most abundant retouched tool in both clusters (Supplementary, Table S4). For the faunal remains, the clusters have been named CF1 and CF2, both being very similar regarding the amounts of remains contained but not in relation to the mean length (Table 5). These clusters also present minimal lengths very similar to those found in the lithic clusters, although with some differences in the maximum and mean lengths (Table 5). Regarding the species, the assemblage is dominated by *Rupicapra rupicapra*, which is the most abundant taxon in both clusters (Supplementary, Table S5).

**Table 5:** Clusters of lithic (CL1, CL2) and faunal remains (CF1, CF2) identified in the Level VII of Amalda I.

	n	length (mm)		
		min.	max.	mean
CL1	409	8	101	21.25
CL2	76	10	77	26.04
CF1	181	10	130	25.74
CF2	191	10	160	23.52

### Directional patterns

**Figure 5:**
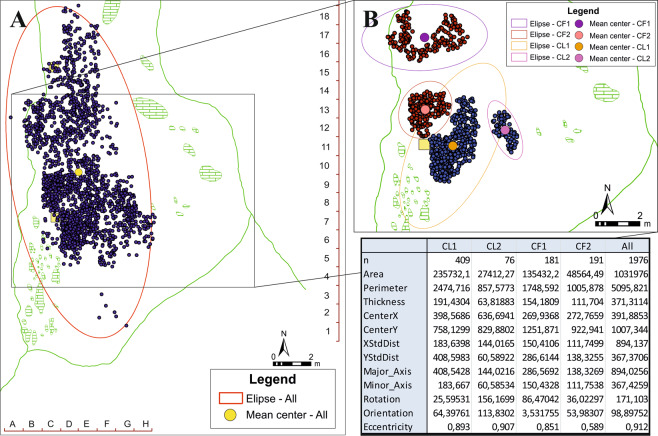
Directional distribution ellipses of the whole assemblage (**A**) and the clusters classified (**B**) in the Level VII, Amalda I.

All the groups, except CF2, show quite high eccentricity values, mainly in the case the whole assemblage (*e* = 0.912). The ellipse CF2 is the most circular, with the lowest *e*-value (*e* = 0.589). In the case of the ellipse orientations, there is no similarity among the groups, although the orientation pattern between the ellipse of CL2 (113.83°) and the ellipse of the whole assemblage (98.89°) are similar (Fig. 5). The CF1 ellipse is practically perpendicular to the cavity (Fig. 5), while the CF2 one tends to the east although the shape is almost circular. The ellipse of the CL1 also tends to the east, in comparison with the ellipse of CL2 (Fig. 5). All these data are very significant, because all the groups, except CL2, show a very different orientation pattern with respect to the axis of the cavity. According to the MBR (*minimum bounding rectangle*) calculation, the orientation of the cave is 175.9°. It is also important to take into account that all the ellipses present very different mutual orientation patterns.

### Non-identifiable faunal remains

The analysis of the distribution patterns according to the taphonomic modification of the non-identifiable faunal remains shows several differences between them, although the main concentrations are generally located in the limits established by Kernel. The remains with gnawing marks are mainly concentrated in the square 11C (Supplementary, Fig. S7), while the digested bones appear in 13C (Supplementary, Fig. S8). In both cases, the application of Getis-Ord Gi* and Anselin Local Moran’s has allowed clear delimitation of the statistically significant accumulation zones, reducing the area of accumulation of remains. Regarding the remains with anthropic alterations, the highest concentration is located in the square 12C (Supplementary, Fig. S9), as in the case of burnt bones (Supplementary, Fig. S10). However, in the first case the remains seem to be more dispersed than for the burnt remains, something that in both cases is reduced when Getis-Ord Gi* and Anselin Local Moran’s I are applied. The statistically significant accumulation of burnt bones is larger than in the remains with evidence of anthropic alteration. The remains with evidence of water dissolution are located in the square 9C, this accumulation being the most concentrated in a specific point of the excavated area (Supplementary, Fig. S11).

## Discussion

**Figure 6:**
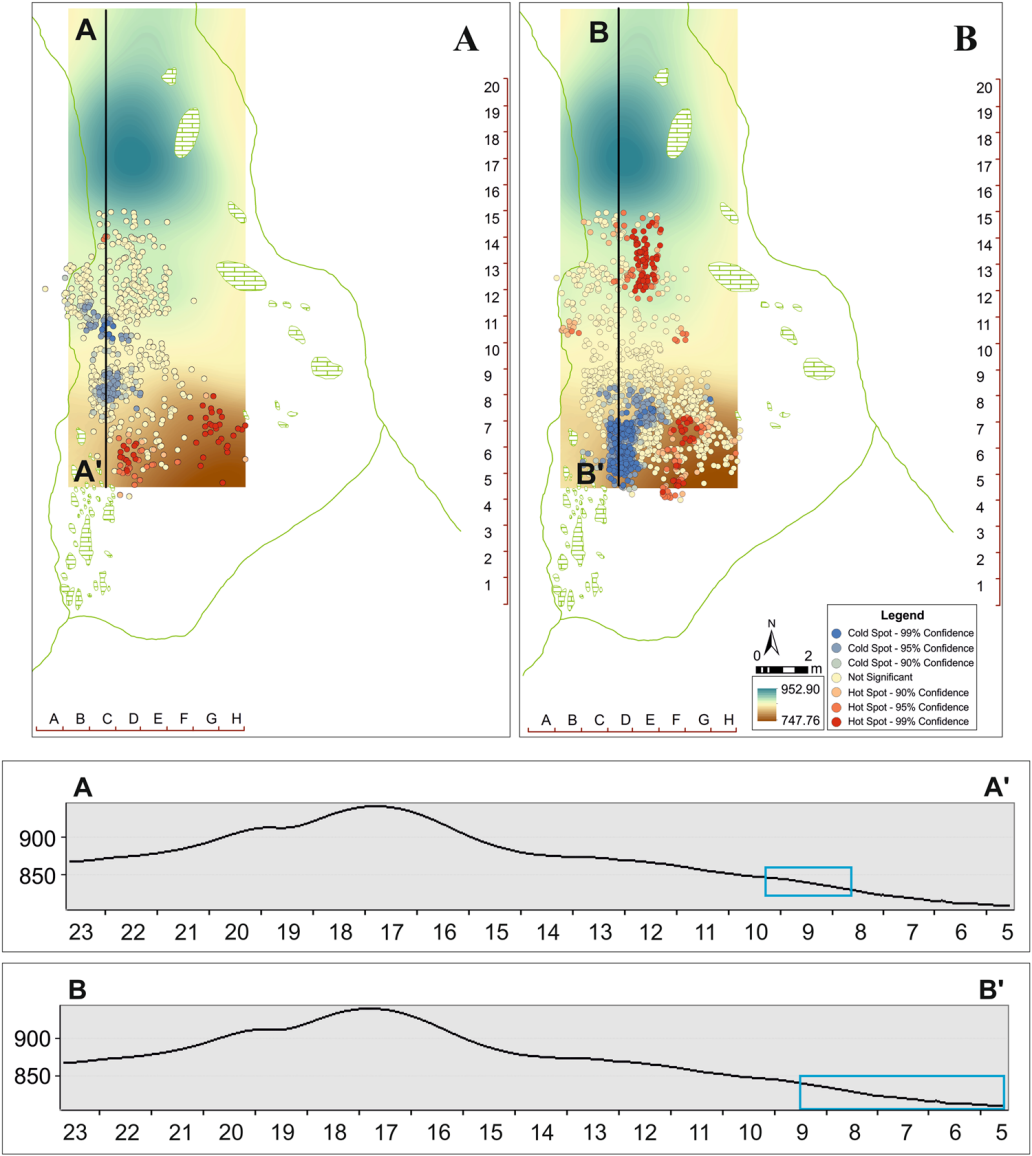
N-S direction of the palaeotopographic profiles that show the Level VII palaeo-relief and the position of the coldspots clusters.

The palaeotopographic reconstruction of Level VII of the Amalda I site shows that the accumulation patterns seem to be independent of the palaeotopography of the level. The densest accumulation area of lithic and faunal remains is regular and with a gentle slope. However, in the lowest zone towards the southeast of the excavated area there are hardly any remains. This pattern is general over the whole site, since the denser areas are located in the central and western part of the site, while towards the east the density of materials is considerably lower. The materials are not accumulated following the slope, nor in the more depressed zone of the palaeotopography (Fig. 6). If the materials had been transported by agents like water, they probably would have accumulated in the more depressed zone. The Getis-Ord Gi* statistic identifies the remains located in this zone as a hotspot, although they are larger than those in the main accumulation. This pattern is the same for faunal and lithic remains, although the sample size in the case of the faunal remains is not significant enough to establish comparisons with the results obtained by Getis-Ord Gi*. As was seen earlier, the lithic materials identified as hotspots are more dispersed and located in the margins of the depressed zone, not in the deeper part. In the case of the faunal remains identified as hotspots, there are fewer and they are found in the lower part of the depressed zone. It is important to remark that the taphonomic analysis does not indicate any kind of evidence of crushing or breaking produced by falls of limestone blocks. Furthermore, the palaeotopographic profiles of Level VII indicate that the coldspot clusters, where the smaller remains are clustered, are not found in depressed zones or with low slope (Fig. 6). The clusters of faunal and lithic remains are in very close spatial positions, even lying opposite each other. If the materials had been transported by water flows, it is reasonable to think that the accumulation of smaller remains would be in the more depressed zone. On the other hand, in this case the smaller remains would have been winnowed out and the larger ones would have remained, so the concentration of smaller items in the nearest margin would not be explicable. Additionally, no evidence of gravels or sand that would indicate the existence of water currents that could transport and select these materials has been observed or documented in this zone of the site^6,19^. That being said, the faunal and lithic remains show a little evidence of alteration by water, as we can see in the use-wear analysis^22,23^ and taphonomy (see Supplementary). However, we cannot know if this alteration by water could have been due to flows (small and ephemeral) or to zones with more humidity, maybe associated to dripping points, something common enough in caves. In the descriptions given in^19^ there are no references that suggest the presence of water flows. Therefore, we can propose that in Amalda I the water effect was not strong enough to winnow or rearrange the spatial distribution of the remains. In addition, it is important to emphasize that the lithic assemblage does not show any sorting or selection by shape or size.

Regarding the spatial patterning, there are elements other than water flows that can alter the arrangement of the archaeological materials and disturb a possible spatial organisation of the occupation area. The presence of carnivores in Amalda I is evident^19–21,33^, a point we can verify not only by the presence of carnivore alterations in bones, taxonomic prey representation, their skeletal and age profiles, but also from the dating results that we have obtained, a fact that could be related to the disturbance activities of carnivores after humans left the cave. The trampling of these animals could have provoked the percolation of remains to the lower levels. However, taking into account the impact of carnivore activity in anthropized spaces^24^, the alteration of the spatial patterning of Amalda I can be described as discrete. The carnivore activity in Amalda I could have been due to attraction by the remains previously left by the humans^19,21,33^. The chance that these activities altered the original position of the remains is very high, and some elements that could offer clues to the existence of structures like hearths could even have disappeared, which would explain the abundance of thermoaltered remains in some points of the occupied areas. However, this possible postdepositional alteration derived from the presence of carnivores would not explain the existence of distinct accumulations of faunal and lithic remains located in spatially different positions, even lying facing each other. If the activity of carnivores had been intense^24^, there would be a high probability that the two well-differentiated areas of higher material concentration would not have been preserved. The site of Abric Romaní has evidence of carnivore access to remains leftover by humans in a zone identified as *toss*^34^, in a zone further from the hearth and where the larger bones were located. In Amalda I, the main accumulation of remains with evidence of carnivore activity is observed around the cluster CF1, which is located in an inner zone, further away from the main accumulations of fauna and lithic remains (Supplementary, Figs. S7 and S8).

Concerning the gap between the main clusters of faunal and lithic remains, this cannot be explained by the excavation process or by the presence of large blocks, which are lacking in this area of the cave. Since the evidence does not suggest that any natural process or recording error could have generated this spatial disposition, other explications can be considered. The well-differentiated spatial distribution and clear clustering of small remains, together with the presence of accumulations of larger remains placed aside from the main accumulation area, recalls the model described by Binford^34^. However, we do not find a mixture of lithic and faunal materials forming a single cluster. A clearly separated accumulation of lithic and faunal remains raises the possibility that this patterning arose from the conduct of parallel and simultaneous activities, such as different resource processing work (butchery, wood working, etc.) and/or knapping. The presence of several individuals carrying out similar or different activities in parallel can generate distributional patterns different from those described by Binford^34^. In the case of Amalda I, the accumulations coincide with the coldspots clusters, with materials no more than 20 mm long. The rest of the identified groups present slightly greater lengths, apart from the fact that their spatial location is clearly different than in other clusters with smaller remains.

This structuration of activities seems to be related with technological organisation in these Neanderthal groups. As previous work has established^22,23^, lithic technology is organised to supply different kind of tools for diverse activities. This has made it possible to identify the use of large mudstone flakes, cleavers and bifaces for heavy activities, such as the initial phases (extraction) of butchering and woodworking; medium-sized flakes and retouched tools made of flint for skin processing or woodworking; and small flint microlithic tools for final phases, such as filleting, sinew cutting, tool maintenance, etc. This structuration of technology, together with that of the occupational space and activities, suggest an organisation of the production that could have involved some kind of social structure.

**Figure 7:**
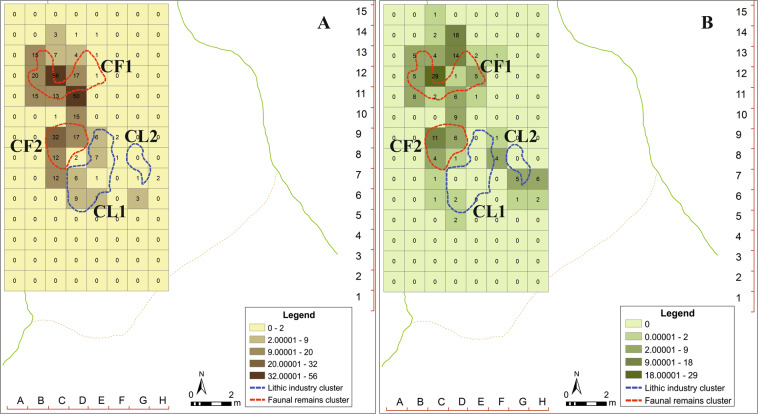
Density maps of burnt remains (**A**) and bones with cutmarks (**B**), overlapped in the main clusters of lithic (CL1, CL2) and faunal remains (CF1, CF2).

The fact that the two main clusters (CF2 and CL1) are separated by a gap with a considerably lower density of remains opens up the possibility of a hearth as a structuring spatial element^35^. Several ethnographic studies have focused on the use of fire and its possible traces at archaeological sites^36–44^, while other authors have centred on the study of those elements that allow intentional fires to be distinguished from fires of natural origin^42,45–51^. While at Amalda I there is evidence of fire use (185 burnt remains according to Altuna’s data^19^, and 340 in the “non-identifiable” assemblage), some of them even calcined^19^, we cannot confirm the existence of hearths, since no ashes, reddened sediments or any kind of basin shape to allow inferring the presence of this type of structures has been identified. The presence of burnt remains is indicative of the processing and consumption of prey, as well as the controlled use of fire^52^. In addition, the calcined bones seem to be an indicator of controlled combustion, where the temperatures reached were high enough to cause the calcination of the remains^46^. Direct exposure can also produce this kind of alteration, either by direct contact or from the use of bones as fuel^14,57–63^. At Amalda I, the larger concentration of burnt bones is located in the squares 12C (n = 56) and 11D (n = 50), according to the data obtained after the analysis of the non-identifiable faunal remains (Fig. 7). In the case of the absence of ashes, this could be due to water effects, such as dripping during the rainiest seasons, causing their disappearance. The burnt remains, dehydrated due to their exposure to the fire, are lighter, so their transport would have been easier as they are less dense^64^. It is important to highlight that exposure to fire can produce greater fragmentation and fragility in these elements^59^, generating accumulations of small remains as a consequence of the exposure. The lack of clear evidence of domesticated fires, such as structured hearths, burnt sediments or reddened stones, could be the result of taphonomic processes rather than the absence of fire itself^52,53^. Not all sites satisfy the necessary and required conditions for the conservation of fire evidence^54^. In these cases, studies focused on the spatial distribution of materials have yielded one of the most appropriate tools for identifying and pinpointing of possible hearths^52,53,55,56^.

The data obtained at Amalda I show that there is a different spatial layout of the main accumulations, not only in relation to the type of material, but also according to the length of the remains. The archaeological record is full of sites with differential spatial distribution patterns, such as Qesem Cave^52,65^, Kebara^66–68^, Abric Romaní^69–80^, *inter alia*), Bolomor^81,82^, El Salt^83,84^, Grotte Vaufrey^85,86^ o Pincevent^87,88^, to name just a few. All these sites have in common the presence of structured hearths, but the absence of this kind of element does not mean that they never existed, since different taphonomic processes can cause their disappearance^52^. Leroi-Gourhan and Brézillon referred to this type of pattern when they coined the term *structures latentes*^87^, defining those archaeological characteristics that can be inferred from the study of the observable spatial distribution patterns of the archaeopaleontological materials. In the case of Amalda I, although there is no direct proof of hearths, we have indirect evidence of fire use, such as thermoaltered remains and their concentration in some points of the cave, mainly in the gap between CL1 and CF2. This could be interpreted as a structuration of the space around a hearth that would have existed in this gap. However, the available data are insufficient to verify this possibility.

The spatial distribution patterns of remains found in Amalda I seem to point to multifunctional areas, where the exploitation of fauna and lithic resources would have taken place in the same area, producing well-defined clusters. Thus, it is possible that these activities were performed at the same time, causing contemporaneous accumulations^75^ according to the activity^35,40,89^. The distribution of the lithic pieces with traces of use also point in this direction, since their locations match the main accumulations and where there are clusters of smaller remains, both fauna and lithic materials. There are also some differences among the clusters identified, because there is a majority of cutting and scraping pieces in the accumulation zone of the cluster CF1, while there is a predominance of pieces focused on cutting in the cluster CL1 (Supplementary, Table S4). The abundance of cutting tools, together with the pieces with direct evidence of butchering activities, indicates that the processing and exploitation for consumption of red deer, bovines, ibex and horse carcasses took place in the cave itself^22,23^.

Lastly, the arrangement of the main accumulations of materials could indicate the existence of a horizontal palimpsest. The reiterated occupation of the cave by humans and carnivores, reflected in the activity traces, would have been seen in several diachronic accumulations, instead of being superimposed one on top of another. Thus, the human occupation is evidenced by the accumulation of lithic materials, prey taxa identified with evidence of anthropogenic modifications, butchering activities documented on the bones and the presence of thermoalterations; while the carnivore occupation is seen in the accumulation of faunal remains, which also could be the result of human activity, since the carnivores could have come to the cave attracted by the remains left by humans^19,21,33^. The alternation in the occupation of the caves by carnivores and humans is amply demonstrated^90–94^, while it is possible to generate horizontal palimpsests that could be wrongly assumed to be the result of the same moment of occupation of the site. Taking this into account, it is possible that most of the archaeopalaeontological assemblage has remained and the main area of activity preserved, but it is important to highlight the fact that the information most susceptible to be transported, like ashes or the smallest remains, could have been lost. The remains with evidence of intervention by carnivores are located in the most distant position and outside the main accumulation zone of humans. In this sense, it could be considered that the carnivores may have accessed the remains left by humans, transporting them to inner and more protected zones, thus prompting another accumulation parallel to the human one.

## Conclusions

The spatial study of Level VII has allowed the unravelling of the event succession that resulted in the formation of the Level, considering the anthropic, carnivore and natural activity. Thus, we have set out the possibility of a space structuration by Neanderthal groups, with the main activity area developed in the external part of the cave, where we find the main concentrations of materials and, thanks to the information provided by bone taphonomy and lithic use-wear analysis, it is possible to infer the type of activities performed. Thus, the clusters CF2 and CL1 seem to indicate the conduct of parallel activities related with resource processing, allowing us to infer a differential deposition of lithic and faunal remains in the same main area of accumulation resulting from the possible task organisation of Neanderthal groups.

The alteration by postdepositional processes of this space of activity has hardly been detected, since two clear areas of accumulation of materials have been perfectly distinguished by the type of material accumulated and the spatially layout of each accumulation. Also, the size of the materials is small, which seems to point to an intense exploitation of resources and the preservation of most of this activity.

Apart from this, an accumulation has been observed in an inner zone of the cave (CF1), where there is a predominance of larger remains. This concentration seems to fit with an accumulation of remains generated by carnivores after the presence of humans in the cave. The absence of successive anthropic and carnivore marks on the same bones, as well as the dates obtained from the bone remains with carnivore marks, dating back to ca. 35-28 uncal BP, reinforce the idea that the carnivore visits were not immediately after the Neanderthal occupation, but when Neanderthals had probably disappeared from this region.

On the other hand, it is important to highlight the degree of alteration by other natural agents, such as water. Although there is evidence of alteration by this agent both in faunal and lithic remains, we have shown that alteration by water was not strong enough to alter the spatial patterning, since water currents have not been detected in the sedimentary record.

In this study, we have combined new spatial analysis tools and palaeotopographic reconstruction of the Level, together with the data provided by bone taphonomy and lithic analysis. All of this information has allowed us to unravel and shed light on the succession of events and processes that acted in the formation of Level VII of the Amalda I cave.

## Material and methods

The spatial study that we present combines the palaeotopographic reconstruction of Level VII with the analysis of the spatial patterns of the lithic and faunal remains. The latter was tackled combining density analysis and hotspots tools to define the empty areas and the clustering. The identified clusters were analysed by archaeological composition, size, shape and direction.

### Data

Several databases have been used to achieve this spatial analysis, both published and unpublished. Regarding the published ones, we have used the existing faunal data obtained by the multidisciplinary team led by J. Altuna during their excavations between 1979-1984, compiled in a detailed monograph^19^. On the other hand, we have also worked with the unpublished databases generated by J. Altuna and K. Mariezkurrena for the faunal remains, and the data of raw material, technology, typology and use-wear for the lithic assemblage produced by J. Rios-Garaizar^23^. Apart from these, we have obtained and included new taphonomic information from the Level VII faunal assemblage, including the non-identifiable fauna that was barely studied in previous analysis^20,21^. In addition, bones modified by carnivores from the Altuna collection and new remains from the recent excavation (2017) were selected for dating to establish the chronology of the carnivore activity in the cave. Regarding to the databases used, there are two types of data: one, materials with XYZ information recorded during the excavation (30%); and two, remains with information about the square of provenance and excavation spit (70%), to which random XY coordinates were assigned following the testing procedure detailed in^23^.

### Palaeotopographic reconstruction

The palaeotopographic reconstruction of Level VII is based on the selection of the set of points with information about the depth (Z coordinate). The interpolation method used was ordinary kriging (8 sectors), in order to produce a high-resolution topography based on the spatial correlation of the data. Thus, we created a continuous and estimated surface to study the relationships between the archaeological spatial distribution pattern and the floor relief during the occupation of the cave.

### Density analysis

The application of Kernel density analysis allows calculating the zones with high or low concentration of elements per unit area, according to the location and spatial proximity among the elements under study^26^. In the case of Amalda I we tested several search radii, selecting a final radius of 0.50 m. Density maps were classified using natural groupings inherent to the data^27^. This classification allowed us to obtain a high-resolution estimation of the zones with a higher concentration of materials, separated from less representative low or medium density zones^28^.

### Hotspots analysis

The spatial study was not only focused on analysing whether the materials were clustered, dispersed or randomly distributed. It also provides the statistical significance of clusters, according to their spatial location and the analysis of the variables that define the groups. In this work, we applied the Getis-Ord Gi* and Anselin Local Moran’s I techniques^29,30^ to analyse the spatial patterns according to the maximum length of the remains and the number of elements in each square (in the case of *non-identifiable* remains) through the quadrat analysis method^95^. To discern the influence of the spatial relationships in the hotspots analysis, we tested the inverse distance, the inverse distance squared and the fixed band, obtaining no significant differences in the results. Additionally, we also tested the FDR correction (False Discovery Rate).

### Directional distribution

Directional distribution and shape of clusters were estimated using the standard deviational ellipse^31^, which measures the trend of a set of points, calculating the standard deviation of the X and Y coordinates from the mean centre to define the axes of the ellipse^32^. This ellipse determines whether the clusters show compact or elongated shapes and their directional trend. Using this, it was possible to analyse the directional features of each group identified for the lithic and faunal remains according to the variable of maximum length. Thus, information was obtained about the standard deviation of the materials that compose each group, both X-axis and Y-axis, as well as the rotation and orientation of every ellipse that the materials of the groups comprise. The data obtained from calculating each ellipse’s geometry has allowed their degrees of eccentricity to be known.

### Taphonomy

For this study, a taphonomic reassessment of the whole faunal assemblage from Level VII was carried out to plot both the anthropogenic and carnivore alterations identified on the ungulate remains spatially. In order to understand the formation of the assemblage and the use of the space use by humans and carnivores during the Middle Palaeolithic, an ArcGIS grid was devised to combine all the taphonomic data associated to the mammal specimens and its spatial location. For the first time, a detailed taphonomic study of the non-identifiable bone fragments is presented, recording the anthropogenic modifications such as cut marks, breakage patterns, thermoalterations, but also the alterations by carnivores, including gnawing and digestion marks.

## Supplementary information

Supplementary Information.
