# Author Correction: New insights for understanding spatial patterning and formation processes of the Neanderthal occupation in the Amalda I cave (Gipuzkoa, Spain)

**DOI:** 10.1038/s41598-020-68761-1

**Published:** 2020-07-22

**Authors:** Laura Sánchez-Romero, Alfonso Benito-Calvo, Ana B. Marín-Arroyo, Lucía Agudo-Pérez, Theodoros Karampaglidis, Joseba Rios-Garaizar

**Affiliations:** 1 Human Evolution Research Center, 3101 Valley Life Sciences Building, University of California, Berkeley, CA 94720 USA; 2 Centro Nacional de Investigación sobre La Evolución Humana, P° Sierra de Atapuerca, 3, 09002 Burgos, Spain; 3 EVOADAPTA Group, Instituto Internacional de Investigaciones Prehistóricas de Cantabria, Universidad de Cantabria (UC), Av. Los Castros, 52, Santander, Spain

Correction to: *Scientific reports*, 10.1038/s41598-020-65364-8 published online 26 May 2020.

The original version of this Article contained an error in the title of the paper, where the space between words “the” and “Amalda” was omitted. This has now been corrected in the PDF and HTML versions of the Article.

