# Supplementary material for: New insights for understanding spatial patterning and formation processes of the Neanderthal occupation in the Amalda I cave (Gipuzkoa, Spain)
Source: Sci Rep. 2020 May 26;10:8733. doi: 10.1038/s41598-020-65364-8 (PMC7250919; doi:10.1038/s41598-020-65364-8)
Supplement: Supplementary file 1 — Supplementary Information. [file 41598_2020_65364_MOESM1_ESM.pdf]

New insights for understanding spatial patterning and formation processes of the Neanderthal occupation in the Amalda I cave (Gipuzkoa, Spain).

Laura Sánchez-Romero[1*], Alfonso Benito-Calvo[2], Ana B. Marín-Arroyo[3], Lucía Agudo[3], Theodoros Karampaglidis[2] and Joseba Rios-Garaizar[2].

[1] Human Evolution Research Center, 3101 Valley Life Sciences Building, University of California, Berkeley, CA 94720, U.S.A.
[2] Centro Nacional de Investigación sobre la Evolución Humana, Pº Sierra de Atapuerca, 3, 09002 Burgos, Spain.
[3] Instituto Internacional de Investigaciones Prehistóricas de Cantabria, Universidad de Cantabria, Santander, Spain.

*e-mail corresponding author: lausanrom@berkeley.edu

SUPPLEMENTARY INFORMATION – section 1

Stratigraphy of the Amalda I cave

In 2017, one of the sections made during the excavations by Altuna's team was opened, in order to review and verify the stratigraphy (1), as well as to identify the levels previously described. The selected study area corresponds to the observations made by Baldeón (2) and Rios-Garaizar (3) regarding the area where Level VII is well preserved. The aperture of this profile allowed us to compile a description of the levels and gave an overview of the whole site, as a starting point for identifying the processes that could have acted in the formation of the deposit. The work performed by the authors' research team took into consideration the stratigraphy described by Altuna (1) as base, as well as the specifications given by Areso et al. (4). The first level we identified in the sequence was Level IX, very characteristic due to the presence of small flattened pebbles of lutite, and Level VIII, sterile reddish clay that rests directly on Level IX. From Level VIII to the top of the sequence, several events were identified (Fig. S1), some of them correlated with the levels described by Altuna (1).

The sequence described has been labelled with Arabic numerals, to distinguish it from the description given by Altuna's team. Thus, the stratigraphic description of the different events identified in the field, except for those upper levels described by Altuna (removed in this part of the site), from the base to the top, is:

1. Limestone substrate.
2. Black crust strongly cemented and crystallised. Thickness: 2-4 cm.
3. Level composed of gravels and small pebbles (the major axis can reach 2-4 cm), elongated and rounded, polymictics (allochthonous origin). Orange, clayed and plastic matrix. Maximum thickness: 20 cm. Not cemented.
4. Level of orange clay, with some small pebbles and fine gravel. Maximum thickness: 100 cm.
5. Level composed of pebbles and elongated rounded fine gravels, polymictics. The maximum axis reaches 7 cm. This level barely contains matrix and is not

cemented. Erosive contact with the clays from the Level 4. Massive. Maximum thickness: 80 cm.

6. Level composed of heterometrical fine gravels, polymictic, flattened and elongated. Clayed matrix, orange, not cemented. Maximum thickness: 25 cm.

7. Level of red-orange clay, with fine gravels and sands. Maximum thickness: 10-15 cm.

8. Level of rounded fine gravels, polymictics, flattened and elongated. The maximum axis reaches 1-3 cm. Not cemented. Maximum thickness: 5 cm.

9. Level of red-orange clay. Maximum thickness: 20 cm.

10. Level of subangular and subrounded limestone blocks (the maximum axis can reach 20 cm). This level barely contains matrix (clayed, orange), but there are polymictic, rounded and elongated clasts. Not cemented. Erosive contact with Level 9. Maximum thickness: 20 cm.

11. Level of fine gravels and small pebbles (maximum axis can reach 3-4 cm), rounded, imbricated, planar, elongated and polymictic. This level barely contains matrix. Not cemented. Maximum thickness: 10 cm. This level rests directly on Level 10. From this event, the levels described by Altuna (1) have been correlated. This level could match the Level IX of J. Altuna, so it can be named as “level 9”.

12. Clayey-silt level, brown and with a maximum thickness 10-15 cm. This event could correspond with the Level VIII of J. Altuna, so it can be named as “level 8”.

13. Silt-clayey level, with very few fine sands and some angular limestone gravels. Brown and not cemented. Lithic industry and macrofauna at the top. Microfauna. Maximum thickness: 10 cm. In this case, several events have been identified, which could be the facies changes in the Level VII described by J. Altuna. This level has been divided in two sublevels, differentiated by lowercase letters, with this one named as “level 7a”.

14. Level composed of limestone blocks and angular limestone clasts. The size varies between 10-25 cm as it approaches the bedrock. This level barely has matrix, which is silt-clayey and brown. Not cemented. Microfauna. Maximum thickness: 10 cm. This level has been named “level 7b”.

15. Level of limestone fine gravels, with clasts of varied size (some can reach 8 cm of maximum axis) and angular. Silt-clayey matrix, brown and not cemented. Microfauna. Maximum thickness: 10 cm. As in the case of the Level VII described by J. Altuna, we have found several events in this level. Thus, this level has been divided into four sublevels, also differentiated by lowercase letters. This one has been named as “level 6d”.

16. Level composed of limestone clasts. Silt-clayey matrix and brown. Not cemented. Microfauna. Maximum thickness: 5 cm. This level has been named “level 6c”.

17. Brown silt-clayey level, with some fine limestone gravels. Not cemented. Microfauna. Maximum thickness: 10-12 cm. This level has been named “level 6b”.

18. Brown silt-clayey level, with fine gravels and small angular limestone clasts. Not cemented. This level contains lithic industry and faunal remains. Microfauna. Maximum thickness: 5 cm. It has been named “level 6a”.

19. Level mainly composed of silt-clayey matrix, brown and with some fine limestone gravels. Not cemented. Maximum thickness: 5-7 cm.

20. Brown silt-clayey level, with planar and angular limestone blocks. The longest axis can reach 15 cm.

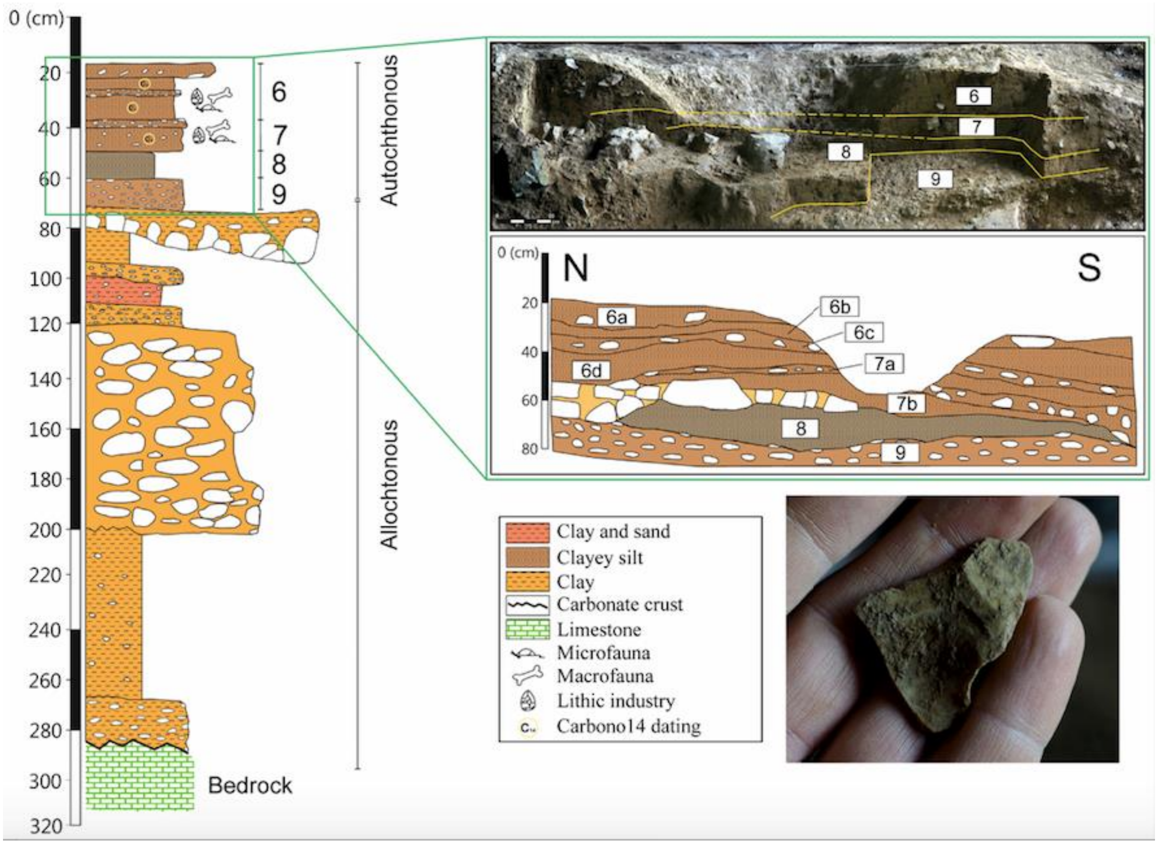

Figure S1: Lithostratigraphic sequence described in the zone of the Amalda I cave excavated in 2017.

SUPPLEMENTARY INFORMATION – section 2

Spatial analysis results

1. Distribution patterns

Regarding the lithic assemblage (n = 1159), X^2 shows a result of 1.31E59 > 43.773, while for the faunal remains (n = 813) it is 3.11E+69 > 37.652; in the case of the whole of the assemblage, the result obtained is quite similar, being 1.55E+66 > 43.773. For K-S, the results also showed a clustered pattern, the value for K-S (96) being higher that the critical value for all the cases: 0.541 > 0.138 for fauna, 0.517 > 0.138 for lithic industry and 0.525 > 0.138 for the whole assemblage. The ANN results indicate that the mean distance for the projected points is clearly less than the expected distance for a hypothetical random distribution (Table S1), indicating that the points are clustered. Furthermore, Global Moran's I also points to a clustered distribution for all the cases analysed (Table S1). The incremental spatial autocorrelation has allowed detection of the distances where there is the maximum clustering, indicating that they coincide with the projection of the lithic industry and all the materials (Table S1). Application of the Ripley's K function has corroborated the clustered nature of the data (Table S1) and has verified whether there are variations in the clustering of the materials in a distance range (10).

	All	Lithic	Fauna
Average Nearest Neighbour (ANN)			
NN observed	8.02	9.46	11.868
NN expected	13.341	12.13	17.649
NN ratio	0.601	0.779	0.672
NN z-score	-33.824	-14.335	-17.745
p value	0	0	0
Ripley's K Function			
Maximum difference observed-expected	122.535	47.496	71.853
Global Moran's I			
Moran's index	0.429	0.535	0.318
Expected index	-0.01	-0.01	-0.01
Z-score	5.893	7.449	4.705
p value	0	0	0
Incremental Global Moran's I			
Maximum peak (m)	2.579	2.579	3.369
Distribution	Unimodal	Unimodal	Unimodal
Z-score	6.824	9.72	4.35

Table S1: Statistical results obtained from the application of ANN, Ripley's K Function, Global Moran's I and Incremental Global Moran's I methods.

2. Definition of the main clusters

Regarding the lithic data, the results obtained applying the fixed band show a clear clustering of coldspots towards the middle part of the cave (Fig. S2), while the two hotspot clusters are located towards the inner part and in a zone closer to the coldspot cluster. The application of the FDR correction hardly changes the distribution and extension of the highlighted clusters. The clusters with high values (hotspots) show a mean length of 33.6 mm (hotspot 1) and 30.5 mm (hotspot 2). Regarding the coldspot

cluster, the mean length is 19.97 mm (Table S2). In all the clusters, the predominant raw material is flint, although there are other materials such as quartz, quartzite, lutite, ophite and mudstone.

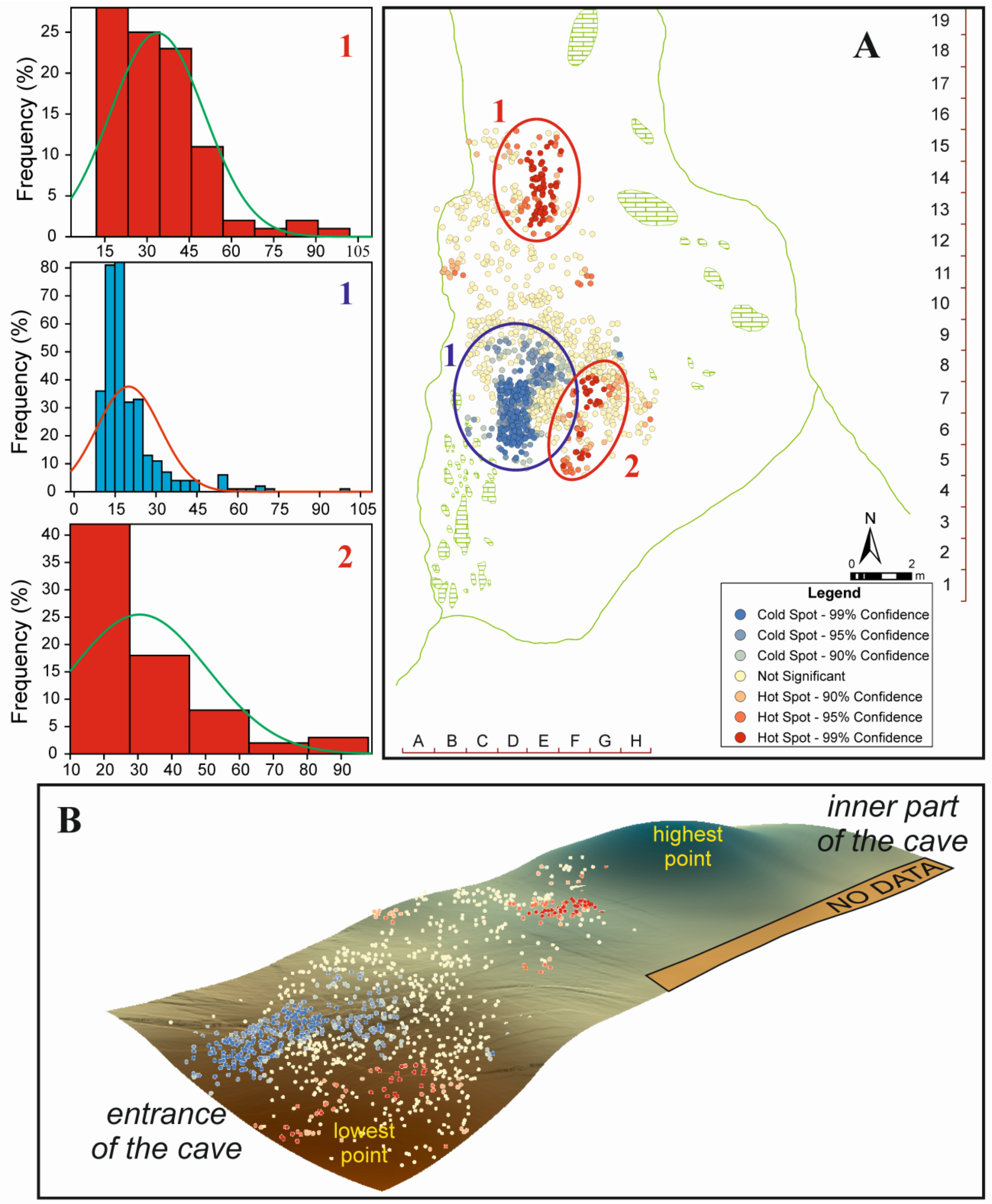

Figure S2: Hotspot classification of lithic industry according to the length (A), and their projection onto the palaeotopographic reconstruction (B) of Level VII, Amalda I.

In the case of the faunal remains, the pattern observed is quite similar to that obtained for the lithic data. The fixed band shows several statistically significant clusters, both low and high values. As regards to the coldspots, they are located towards the inner part of the cave, while the hotspots are in an outer area, closer to the cave entrance (Fig. S3). Coldspot 2 is located just above the lithic coldspot cluster. Neither coldspot clusters are composed of a large amount of materials, especially compared with the clusters obtained

in the lithic analysis. The two hotspot clusters also comprise a fairly small number of remains, one of them (hotspot 1) almost vanishing when the FDR correction is applied (Fig. S3). This also happens with the main coldspot cluster. Regarding the mean length of each cluster, the coldspots vary widely. Coldspot 1 shows a mean length of 25.5 mm, while for coldspot 2 this is 33.6 mm (Table S2). It is important to bear in mind that this difference could be due to the sample size of the clusters, which makes the two accumulations difficult to compare. Something similar is observed in the hotspot clusters. Hotspot 1 shows a mean length of 38 mm, while for hotspot 2 the mean rises to 68 mm. When the Getis-Ord Gi* result is projected onto the palaeogeotopography of Level VII is possible to observe that the coldspot cluster coincides with the densest zone highlighted by the kernel analysis. This cluster is located in an intermediate zone with little slope. The sparser coldspot cluster is further in, in a slightly more elevated zone of the cave. On the other hand, hotspot cluster 1 is in a lower position and where the slope seems to be more regular. In the case of hotspot cluster 2, this is in the more depressed zone of the palaeotopography (Fig. S3).

FAUNAL REMAINS			
**Cluster**	**n**	**Mean (mm)**	**Stdev**
Coldspot 1	37	20	16,765
Coldspot 2	77	24,026	28,976
Hotspot 1	32	35,781	25,406
Hotspot 2	29	58,621	34,812
LITHIC REMAINS			
**Cluster**	**n**	**Mean (mm)**	**Stdev**
Coldspot 1	320	19,978	11,693
Hotspot 1	93	33,677	16,759
Hotspot 2	73	30,562	20,128

Table S2: Number of remains, mean length and standard deviation of the remains contained in each cluster classified by Getis-Ord Gi* according to the length.

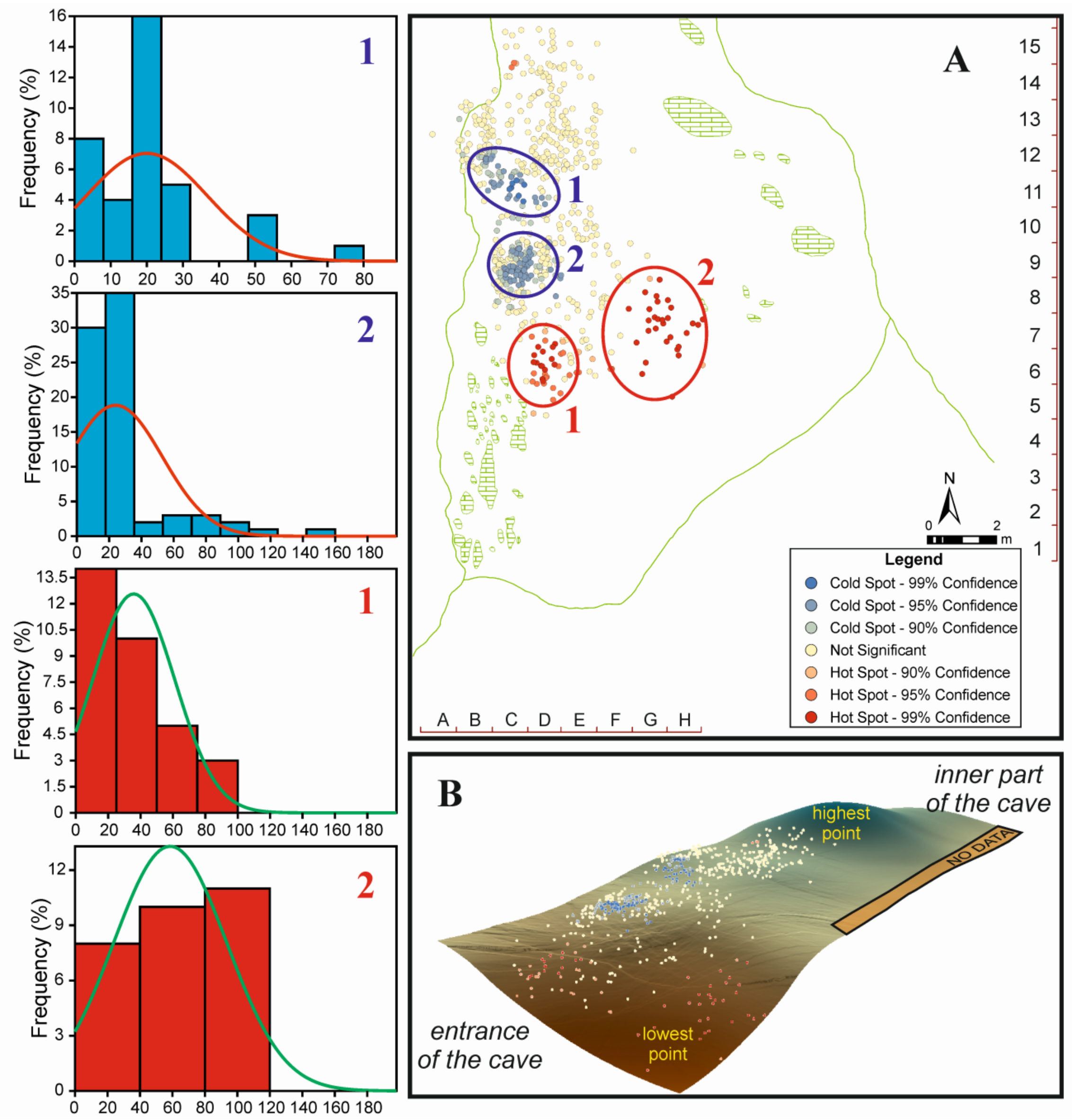

Figure S3: Hotspot classification of fauna according to the length (A), and their projection onto the palaeotopographic reconstruction (B) of Level VII, Amalda I.

Regarding the application of the Anselin Local Moran´s I, the projection of the lithic remains according to the fixed band shows a clear concentration of low values (light blue) close to a scattering of red points (Fig. S4), which indicates that they are high values but surrounded by low values. It indicates a significant concentration of mainly low values. Near to this cluster, towards the east, it is possible to observe a change in the concentration pattern, where there is a predominance of dark blue and pink points, which indicates a concentration of high values. This pattern is the same that we find in the accumulation located towards the inner part of the cave, where we observe a predominance of dark blue points (Fig. S4). This colour indicates low values but surrounded by high values, while the pink colour indicates high value clusters. In this case, there seems to be a predominance of high values in this zone of the cave. The application of the FDR correction does not delete relevant information about the concentration highlighted by Anselin Local Moran’s I. In the case of the faunal remains, the application of the FDR correction shows statistically not-significant distributions. The fixed band shows two zones clearly differentiated. One of them is by the cave entrance and dominated by dark

blue and pink points. The other zone, in an inner part, shows a predominance of high values surrounded by low values (red), although to the south the pattern changes and there is a majority of clusters of low values (light blue) (Fig. S4).

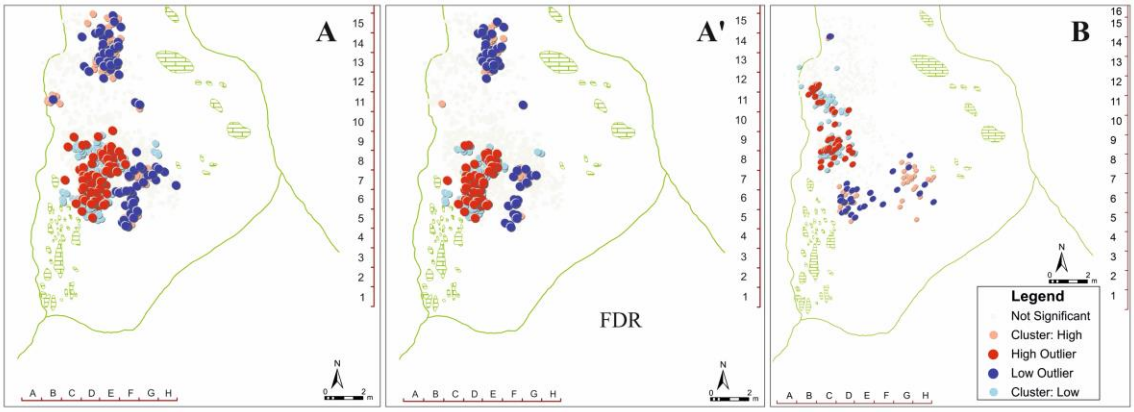

Figure S4: Anselin Local Moran's I lithic distribution map with (A) and without (A') the application of the FDR correction. This analysis has been also applied to faunal remains (B).

The patterns obtained after the application of the Anselin Local Moran's I to the lithic and faunal remains according to the maximum length have allowed us to detail the clusters identified in Amalda. The zone defined by the kernel density as the maximum concentration of remains (both lithic and fauna), is dominated by smaller remains. However, in this area there are also remains longer than the dominant length in the accumulation here. Furthermore, the longer lithic remains are mainly located in the cluster located towards the inner part of the cave and in another cluster that is adjacent to the main concentration of materials (coldspot). Both clusters present greater lengths, although there are also smaller remains. In the case of the fauna, there is a similar pattern. The maximum concentration zone seems to be dominated by smaller remains (low values), while the other clusters (defined by Getis-Ord Gi* as hotspots) show larger remains (high values).

The application of Getis-Ord Gi* and Anselin Local Moran's I to the density values has allowed us to verify the statistical significance of the main accumulations of lithic and faunal remains (Fig. S5). To do so, we have applied the fixed band and Euclidean distance, as well as the FDR correction, to adjust the p-values to bound the statistical confidence more tightly. The result obtained has allowed the main groups of accumulation of lithic and faunal remains to be delimited according to statistical criteria. Thus, with a minimum degree of confidence of 95%, the main groups of materials have been defined for the Amalda I site. When the delimitation of the groups is superimposed onto the maps obtained with the kernel density analysis, it is possible to observe that the main accumulations of materials do not coincide spatially, but stand practically opposite one another (Fig. S5).

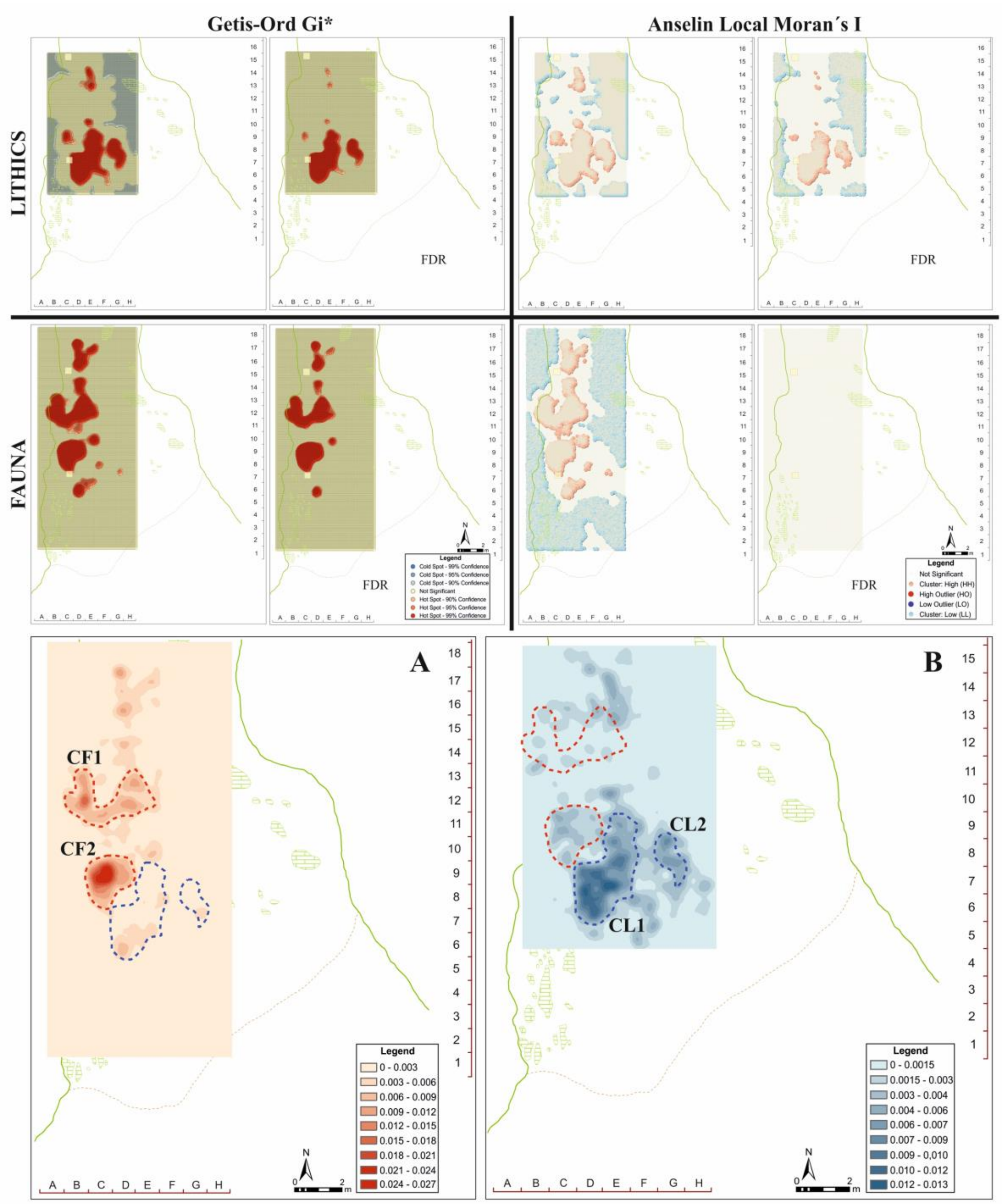

Figure S5: Getis-Ord Gi* and Anselin Local Moran’s I applied according to the density data. Clusters identified for fauna (A) and lithic (B) remains in Level VII, Amalda I.

3. Characterization of the groups

Although the most abundant raw material is flint (n=311), there are other many materials less well-represented, such as quartz, quartzite, mudstone or ophite. The same percentage observed in the overall assemblage is repeated in the clusters identified. Regarding the support, the most common pieces are splinters and fragments (n=83), as well as microflakes (n=63), flakes (n=61), reshaperning flakes (n=60) and *outrepassé* flakes (n=52). In the case of the tools, the cluster is dominated by non-retouched (n=353), such as flakes and microflakes (Table S4).

Support	CL1	CL2
Splinter	2	
Fragment	83	9
Blade	4	3
Bladelet	12	6
Flake	61	17
Resharpening flake	60	7
Cortical flake (1 & 2)	47	12
Outrepassé flake	52	9
Kombewa	13	2
Overshot flake	8	1
Microblade	63	7
Core	4	3

Tool	CL1	CL2
Buril	2	
Chopping tool	1	
Non-retouched	0	1
Denticulate	9	
Cleaver	4	
Retouched blade	1	
Retouched bladelet	1	
Notch	1	1
Bladelet core	1	
Flake core	3	3
Step retouched piece	4	1
Retouched	7	4
Mousterian point	1	
Rabot		1
Sidescraper	20	7
Scraper	1	
Non-retouched (flakes and microflakes)	353	57

Table S4: Lithic tools and supports for the clusters CL1 and CL2 of the Level VII, Amalda I.

However, among the retouched tools, there is a predominance of sidescrapers (n=20) and denticulates (n=9). As in the case of CL1, the most abundant raw material is flint (n=59). The most common supports are flakes (n=17) and cortical flakes (n=12), while in the case of the tools there is a predominance of non-retouched (n=57). In general terms, there is a great variability in the shapes (Fig. S6), in spite of the clear differences in the sample size of the clusters. Most of the pieces show a tendency to rectangular and quadrangular forms, but narrow, although there are some thicker pieces. It is important to highlight the small number of elongated pieces, even in the case of the most numerous cluster (CL1). The pattern observed for both clusters is expectable for a Middle Palaeolithic lithic assemblage.

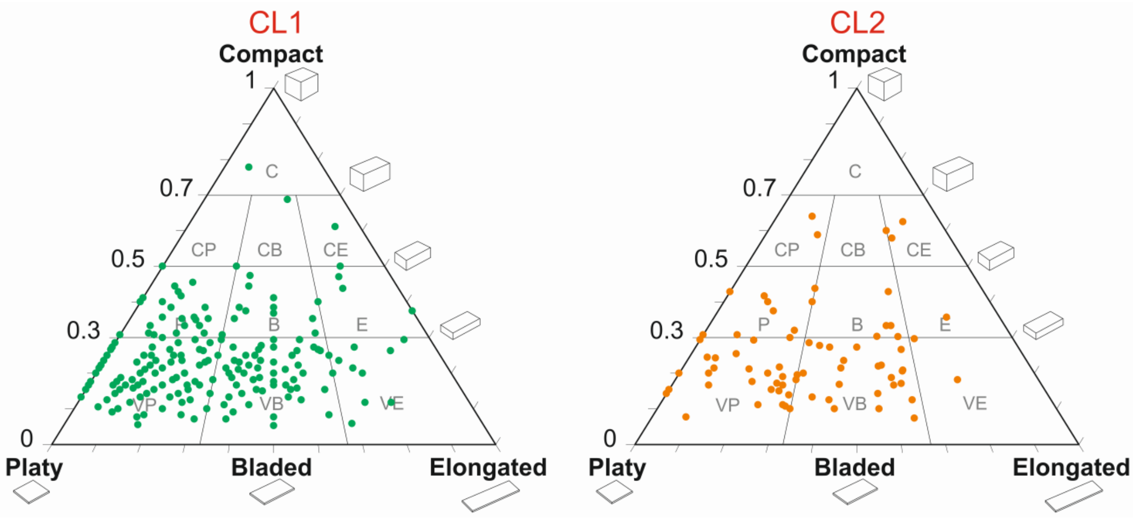

Figure S6: Shape patterns for the lithic remains in the CL1 and CL2 clusters.

As regards the species, there is a predominance of *Rupicapra rupicapra* remains (n=98), as well as *Bos primigenius* (n=21) and *Cervus elaphus* (n=20) (Table S5). As for carnivores, the most common is *Vulpes vulpes* (n=15), although there are also remains of *Canis lupus* (n=4) and *Ursus spelaeus* (n=7). There is a clear abundance of teeth (n=34) and, with respect to the axial skeletal parts, a predominance of ribs (n=16) and vertebrae (n=20). In the case of the cluster CF2, *Rupicapra rupicapra* is again the most abundant taxon (n=125), while the number of carnivores is quite small compared to CF1 although with a wider variability: *Panthera pardus* (n=1), *Canis lupus* (n=2), *Ursus spelaeus* (n=3) and *Vulpes vulpes* (n=1) (Table S5). As in the case of CF1, the most abundant skeletal part is teeth (n=36), as well as ribs (n=25) and vertebrae (n=24).

The selected groups are mainly composed of small remains, both for CL1 (21.25 mm) and CL2 (26.04 mm). In both cases, the minimum length is at most 10 mm. The fauna clusters show minimum lengths very similar to those we found for the lithic groups, while the maxima are higher than for the lithic remains. Regarding the mean lengths, the fauna groups are slightly longer, at 25.74 mm for CF1 and 23.52 mm for CF2. However, it is important to keep in mind that both clusters contain fewer remains than the lithic clusters. It is also necessary to characterise the space, apparently empty, between CF2 and CL1. Here, 17 remains have been documented, with a mean length of 22.47 mm (16 flints and 1 mudstone). All the remains are non-retouched, except 2 sidescrapers. In the case of the faunal remains, 7 remains have been identified (5 of *Rupicapra rupicapra*, one of *Ursus spelaeus* and one of *Capra pyrenaica*) and with a mean length of 20 mm. The data show that this space between the two main accumulations of materials is not the result of an error; several remains have been recorded, although in a considerably lower proportion than the other two clusters that delimit it. In addition to these characteristics, all the modelling and simulations carried out in order to observe the variations in the location of the remains made by k-means (3), show that this space is delimited in all the cases. The slight variations demonstrate that this space contains far fewer remains and this is the same in all the simulations.

**Axial**		**CF1**	**CF2**
	Ribs	16	25
	Vertebrae	20	24
**Appendicular**			
	Scapula	1	1
	Pelvis	3	6
	Calcaneus	1	1
	Carpal	4	7
	Centrotarsal		2
	Phalange	26	28
	Femur	7	13
	Humerus	4	7
	Metacarpus	9	4
	Metapod	1	2
	Metatarsal	8	4
	Patella	6	9
	Radius	2	3

	Sesamoid	5	5
	Talus	4	3
	Tarsal	1	
	Tibia	14	3
	Ulna	6	3
**Cranial**			
	Skull	6	3
	Horn	1	
	Teeth	34	36
	Mandible	1	2
	Hyoid	1	

Table S5: Skeletal parts identified in clusters CF1 and CF2.

4. Non-identifiable faunal remains

The results obtained show that there are slight variations in the distribution of remains according to the taphonomic features, though always inside the densest zone classified by the kernel analysis in the identifiable bones. The identification of the statistically significant clusters was accomplished using the Getis-Ord Gi* and Anselin Local Moran's I statistics, with the fixed band as spatial relationship and with/without the application of the FDR correction. In the case of the Anselin Local Moran's I, the analyses were conducted applying 99 permutations.

The bones with evidence of **alteration by carnivores** (gnawing, scores, punctures) are mainly accumulated in the square 11C (n=63). In relation with the application of Getis-Ord Gi*, the fixed band shows that the main concentration with 99% confidence level is in the squares 10C, 11B-C, 12C-D and 13D (Fig. S7). However, after the application of the FDR correction, the cluster is reduced to the squares 10C, 11C-D and 12C. Regarding the Anselin Local Moran's I statistic, the results obtained show well-delimited clusters which are mutually coherent. The main cluster is located in the squares 11B-C-D, 12C and 13D (Fig. S7), while the squares 10C, 11E and 12D (dark blue) indicate that there is a concentration of low values surrounded by high values. This pattern would represent a statistically significant accumulation of high values around a low value square.

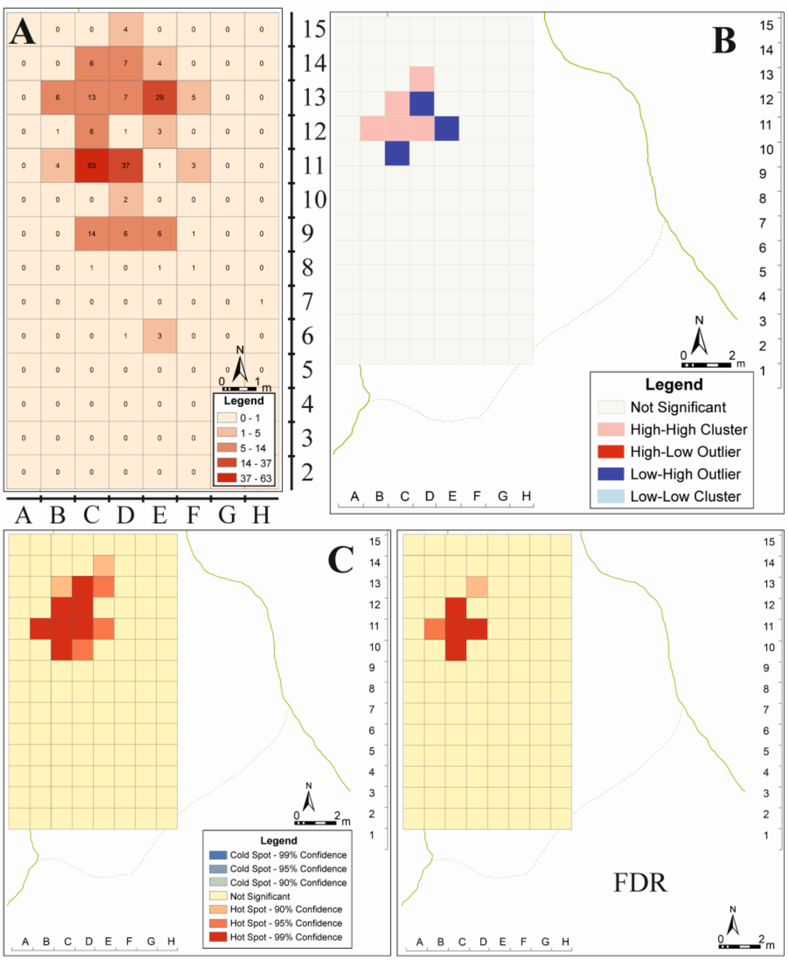

Figure S7: Distribution map by squares corresponding to gnawing marks (A); distribution map according to the application of Anselin Local Moran's I (fixed band) (B); distribution map after the application of Getis-Ord Gi* (fixed band) (C) and with the FDR correction (D).

In the case of the **digested bones**, the most important concentration is in the square 13C (n=200), as well as in the squares 12C, 14D and 9C (n=100). The Getis-Ord Gi* statistic has revealed that the squares 12C, 11D and 9D have the highest confidence levels, while the squares 12B-D and 9D show lower percentages of confidence (Fig. S8). The FDR correction removes these squares, maintaining the others with highest confidence level without any reduction of its percentage (except in the case of the square 9C). The cluster delimited by the Anselin Local Moran's I statistic is located in the squares 11C, 12C-D and 13B-C-D, although it is possible to observe an isolated square (9D) identified as *HH* (Fig. S8).

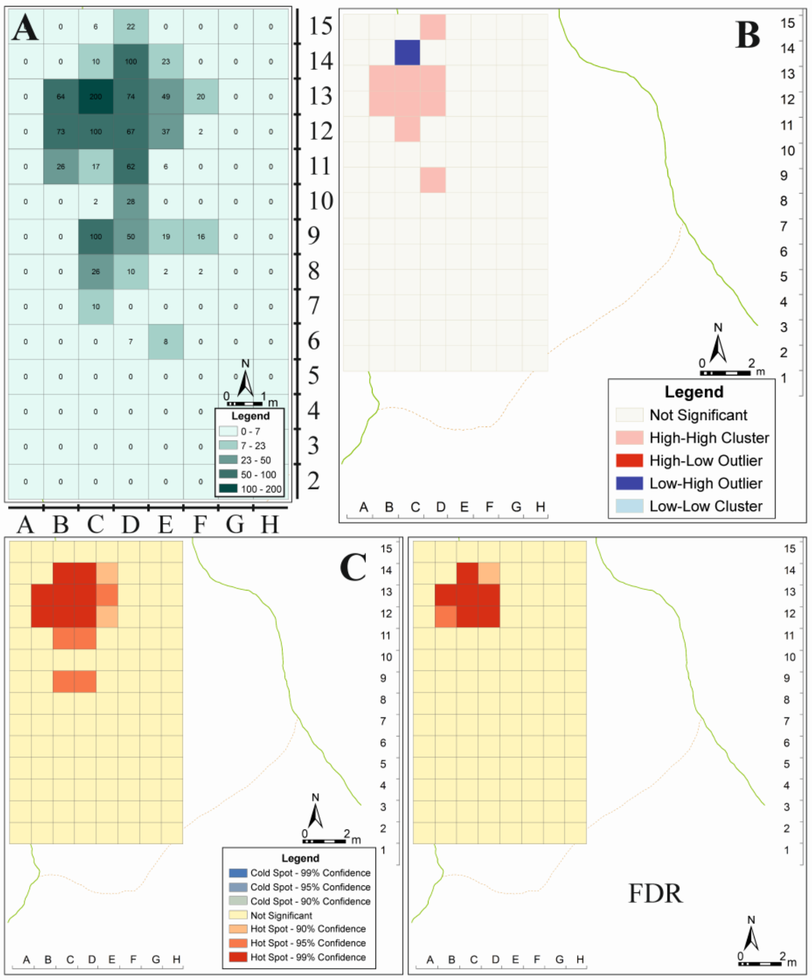

Figure S8: Distribution map by squares corresponding to digested remains (A); distribution map according to the application of Anselin Local Moran's I (fixed band) (B); distribution map after the application of Getis-Ord Gi* (fixed band) (C) and with the FDR correction (D).

The main accumulation of bones with evidence of **anthropic alteration** (fresh fractures, cutmarks and bone flakes) is in the square 12C (n=29), although the rest of the squares also include bones with these taphonomic features like 14D (n=18), 13D (n=14) and 9C (n=11). The application of the Getis-Ord Gi* statistic shows that there are several hotspot squares with high confidence levels, such as 11C, 12B-C-D, 13C-D and 14D (Fig. S9). The rest of the squares also present statistically significant levels, but in lower percentages. These squares disappear when the FDR correction is applied, the squares 11C, 12B-C-D and 13C-D with a 99% confidence level remaining. The square 14D is reduced to 95%. Regarding the Anselin Local Moran's I statistic, there are several squares with high values (11C, 12B, 13C-D and 14C) and three squares that reflect low values surrounded by high values (10C, 12D and 15D) (Fig. S9).

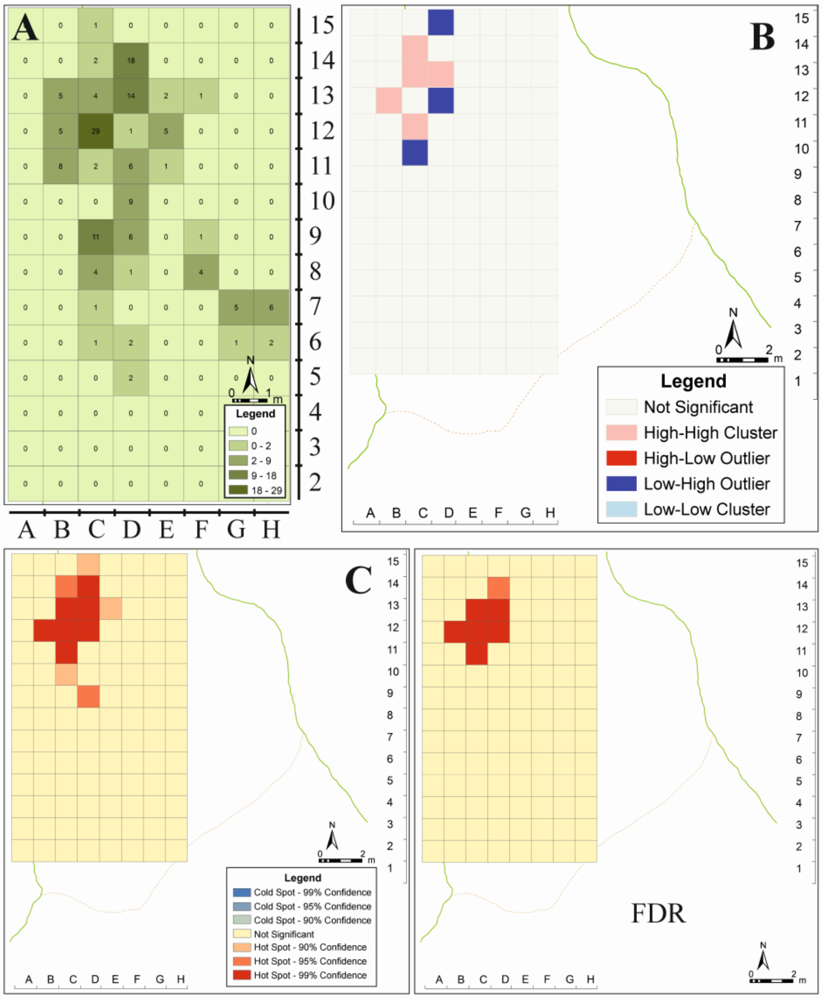

Figure S9: Distribution map by squares corresponding to cutmarks (A); distribution map according to the application of Anselin Local Moran's I (fixed band) (B); distribution map after the application of Getis-Ord Gi* (fixed band) (C) and with the FDR correction (D).

In the case of the **burnt bones**, the main accumulation is located in the squares 12C (n=56) and 11D (n=50). The rest of the squares also contain bones with this kind of alteration but in much smaller proportion. The Getis-Ord Gi* statistic shows several squares with high confidence levels, such as 9D, 10D, 11C-D, 12B-C-D and 13C. The squares 10C, 9C, 8C and 11B-E have lower confidence percentages, and disappear when the FDR correction is applied. The squares 10D, 11C-D, 12B-C-D and 13C are maintained, while the square 9C shows a reduction in its confidence level (Fig. S10). The application of the Anselin Local Moran's I statistic shows a well-delimited cluster, which corresponds to high-value squares: 9D, 10D, 11C-D, 12B-C-D and 14C. The square 10C reflects the presence of a low value surrounded by high values (Fig. S10).

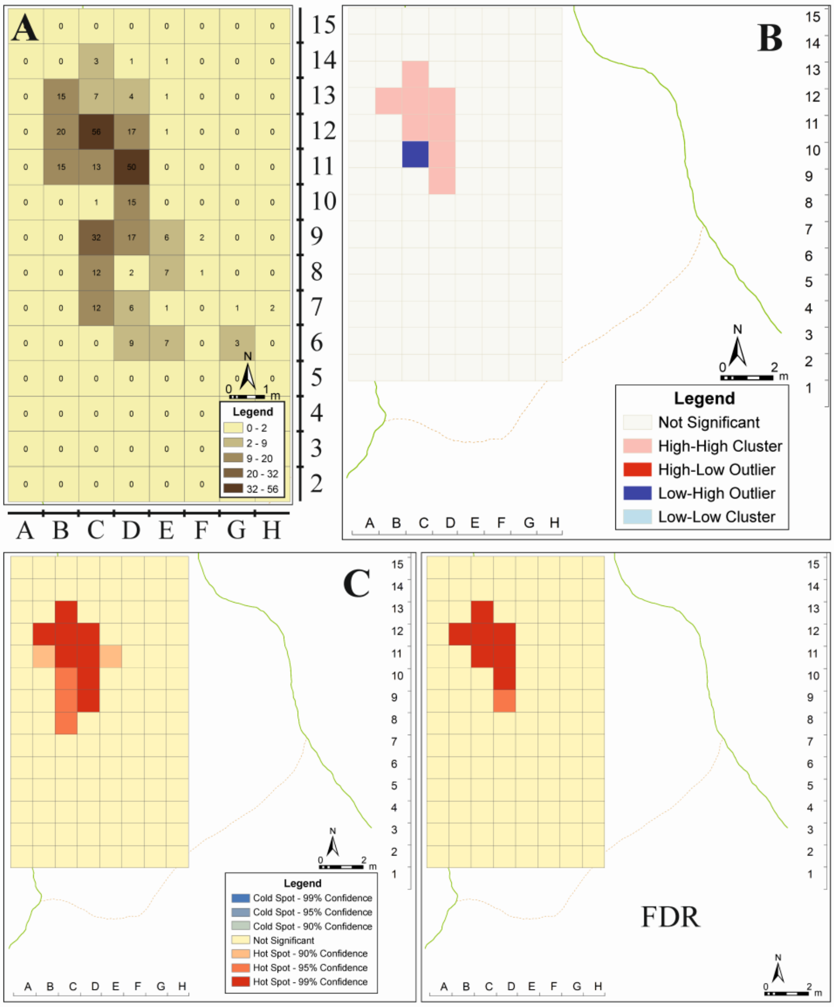

Figure S10: Distribution map by squares corresponding to burnt remains (A); distribution map according to the application of Anselin Local Moran's I (fixed band) (B); distribution map after the application of Getis-Ord Gi* (fixed band) (C) and with the FDR correction (D).

The bones with evidence of **dissolution by water** are mainly clustered in the square 9C (n=300), without any similar concentration in other squares. In this case, the bones are clearly accumulated in a specific zone of the site. The application of the Getis-Ord Gi* statistic shows the same result with and without the application of the FDR correction. Thus, the squares with a confidence level of 99% are 8C, 9B-C-D and 10C (Fig. S11). In the case of the Anselin Local Moran's I statistic, there is a majority of *LH* squares (9B, 10D and 12D) separated from each other (Fig. S11). There are two high-value squares, which are adjacent (8C and 9D).

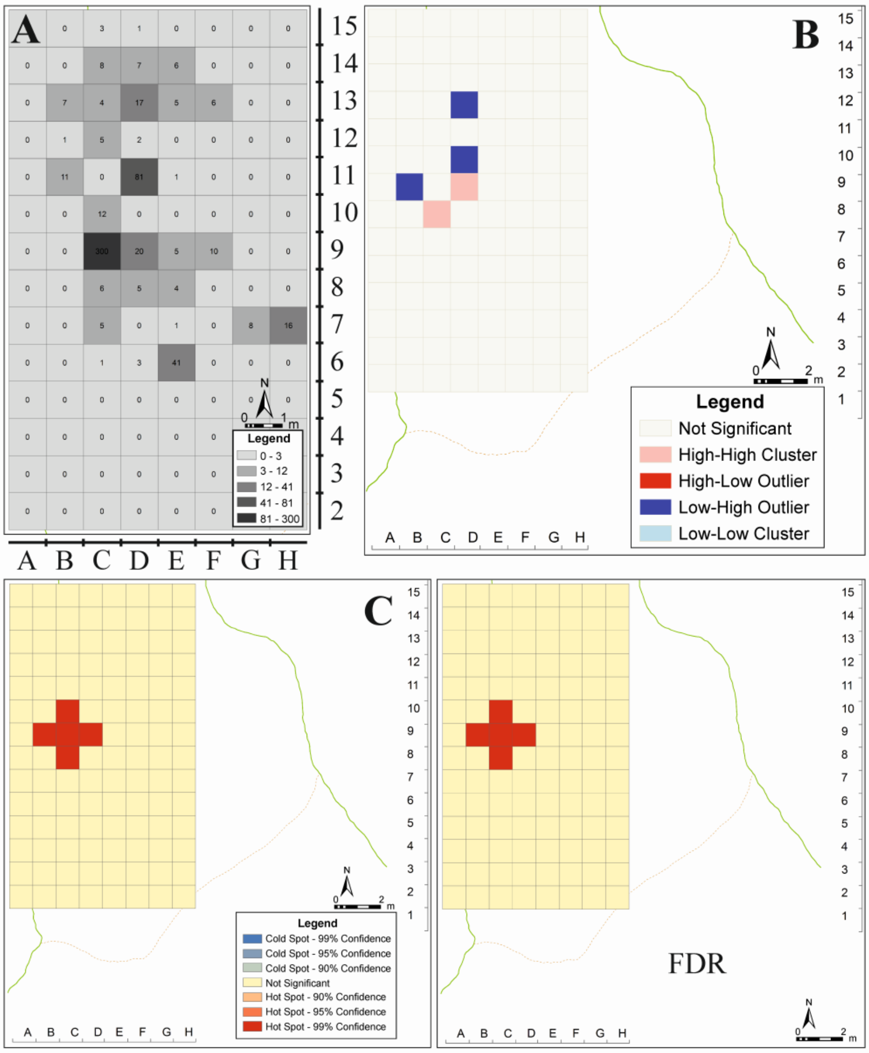

Figure S11: Distribution map by squares corresponding to dissolution by water (A); distribution map according to the application of Anselin Local Moran's I (fixed band) (B); distribution map after the application of Getis-Ord Gi* (fixed band) (C) and with the FDR correction (D).

The densest accumulation of **NISP** is in the square 12C (n=700), followed by 11D and 9C (n=500). This high concentration at this point of the site coincides with the zone of the main accumulation of bones with anthropic activity, both burnt remains and cutmarks, fresh percussion and bone flakes. In addition, this square is adjacent to the zone of the main concentration of remains with evidence of alteration by carnivores, as well as digested bones. The highest confidence level after the application of the Getis-Ord Gi* statistic (99%) is in the squares 9C-D, 10D, 11C-D, 12B-C-D and 13C-D (Fig. S12). The rest of the squares are also statistically significant but with lower percentage values: 8C, 10C, 11E and 13B. The FDR correction maintains the main hotspot squares, except in the case of 9C and 10D, whose confidence percentage is reduced. As in other taphonomic features, the squares with lower confidence levels vanish after the application of the FDR correction. The application of the Anselin Local Moran's I statistic shows a majority of high-value squares (9D, 10D, 11C-D, 12B-C-D and 13C-D), two squares identified as *LH* (10C and 11E), and one square (3D) that indicates a concentration of low values (*LL*) (Fig. S12).

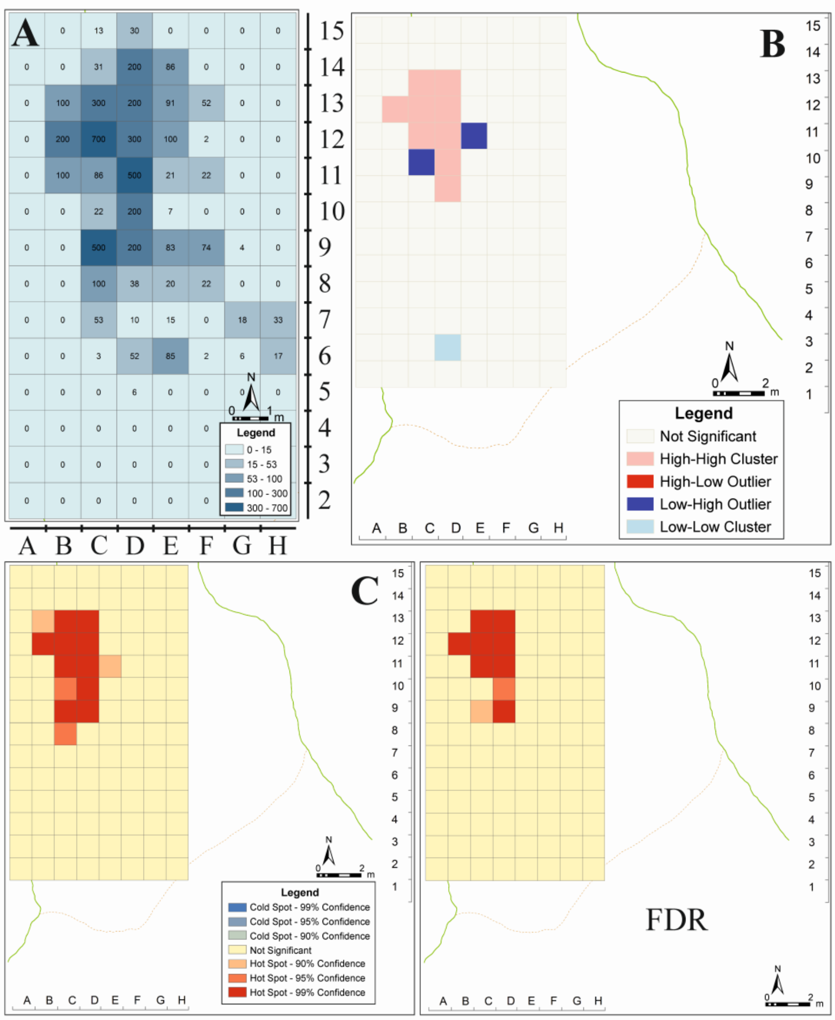

Figure S12: Distribution map by squares corresponding to NISP (A); distribution map according to the application of Anselin Local Moran's I (fixed band) (B); distribution map after the application of Getis-Ord Gi* (fixed band) (C) and with the FDR correction (D).

As in the case of the distribution patterns identified by Getis-Ord Gi*, the Anselin Local Moran's I statistic has identified certain coincidences in relation to the accumulation of remains in certain squares. All the squares show clusters of high values (*HH*) and some of have with different taphonomic features in the same square. The square 11C comprises remains with evidence of digestion and burnt remains, as well as anthropic (cutmarks, fresh fractures, flakes) and carnivore (gnawing, scores, punctures) alterations. The square 11D contains burnt bones and also alterations by carnivores. The square 13D includes remains altered by carnivores, digested and with evidence of anthropic activity, while 13C contains digested remains and bones with anthropic marks. The square 12C comprises remains with alterations by carnivores, digested and burnt remains. Finally, 14C contains burnt remains and also evidences of anthropic activity. The most significant characteristic to highlight is the coincidence in several squares of burnt remains together with bones showing signs of carnivore alteration (gnawing, scores, punctures).

5. Use-wear analysis

The traceology study carried out with the lithic materials of Amalda I (11) has provided very interesting data on the activities undertaken in the cave during the period of occupation (11,3). Most of the pieces seem to show a correlation with the main accumulations of materials (CF1, CF2, CL1 and CL2). The cluster CF1 and surroundings

show a majority of pieces with traces of scraping (n = 5), as well as 5 with evidence of cutting (4 in an undetermined material and one in hard or semi-hard material), one altered, one piece identified as projectile and one with signs of having been used for percussion (Fig. S13). In the case of the cluster CF2, 3 pieces have been found with traces of cutting (undetermined material), one altered and one with traces of scraping (soft material or skin). Between the clusters CF2 and CL1 there are 3 lithic pieces: one with evidence of alteration and two more with traces of cutting (soft and undetermined materials). The cluster CL1 is where there are most pieces with traces of cutting (n = 6) and scraping (n = 3) in undetermined material, as well as 4 altered pieces.

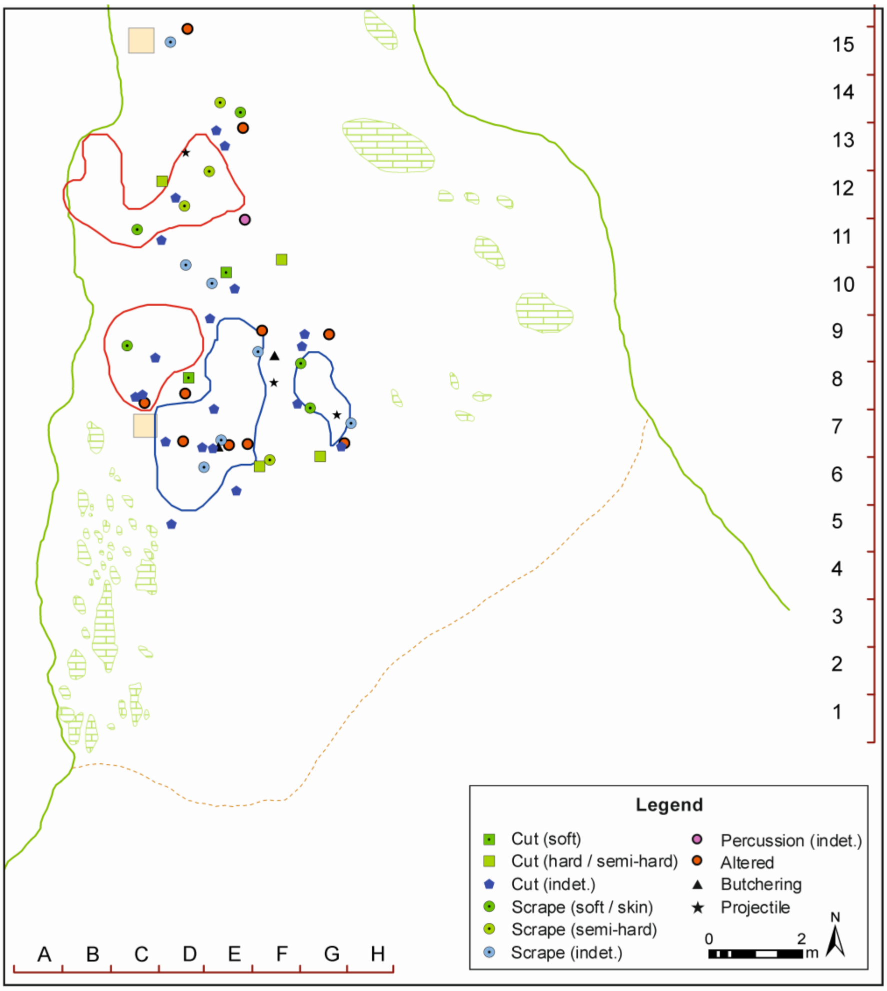

Figure S13: Distribution map of the pieces with identifiable use-wear traces.

SUPPLEMENTARY INFORMATION – section 3

Taphonomic results

We excluded material from squares at the excavation (bands) numbered below 5 and above 15 from our analysis. Those areas were considered unclear stratigraphically. In Altuna's publication (1) a total of 7,340 fragments (88.4% of the assemblage) were classified as indeterminate. Even so, Yravedra (5-7) carried out a later taphonomic study aiming to determine the role that carnivores played in the accumulation and identify the main carnivore accumulator of the chamois, and he focused on the identified ungulates and carnivores and only 54% of the non-identifiable assemblage (3,938 fragments, see Yravedra (6)) initially studied by Altuna, but the indeterminate fragments as a whole did not receive as much as attention apart from a re-quantification and a taphonomic classification, although only of bones classified as large to small in size. In fact, some of the non-identifiable fragments analysed in this study were still unwashed, just as they had been recovered during excavation, and curated at the Gordailua Center for the Cultural Heritage of Gipuzkoa (Basque Country). We washed them all and looked at them individually to provide a general taphonomic study of the whole assemblage.

Regarding the results obtained in this work, our reassessment agrees with the previous taphonomic studies concerning to the identified ungulates and carnivore specimens accomplished first by Altuna (1) and later by Yravedra (5-7). The assemblage used does not include terrestrial and marine gastropods and microfauna remains. The average length of the fragments is 0.6 cm, which indicates an extreme fragmentation of the assemblage. Despite this limited size, several studies (8-9) reveal the importance of these fragments in understanding the formation of the deposit and identifying possible disturbances during and after the level was formed. The cutmarks identified in non-identifiable remains were found on bones with an average length of 3.3 cm, mostly belonging to indeterminate taxa (42%), large mammals (40%), medium-sized (17%) and small mammals (2%). The flakes, produced as a result of bone marrow extraction, have an average length of 1.5 cm and 34% belong to large mammals, 14% to medium-sized mammals and only 5% to small mammals, while for 48% was impossible to distinguish their taxonomic category. Thermoalterated bones were of 0.5cm long on average, 1% with brown colouration, 61% black, 1% brown-black, 33% black-white, 1% grey and 4% white in colour. Regarding the activities of non-humans (Fig. S14), the taxonomy of the digested bones is difficult to pin down, although 17% of them could be identified at least as to mammal size, and of these, 12% could be attributed to the size of a chamois.

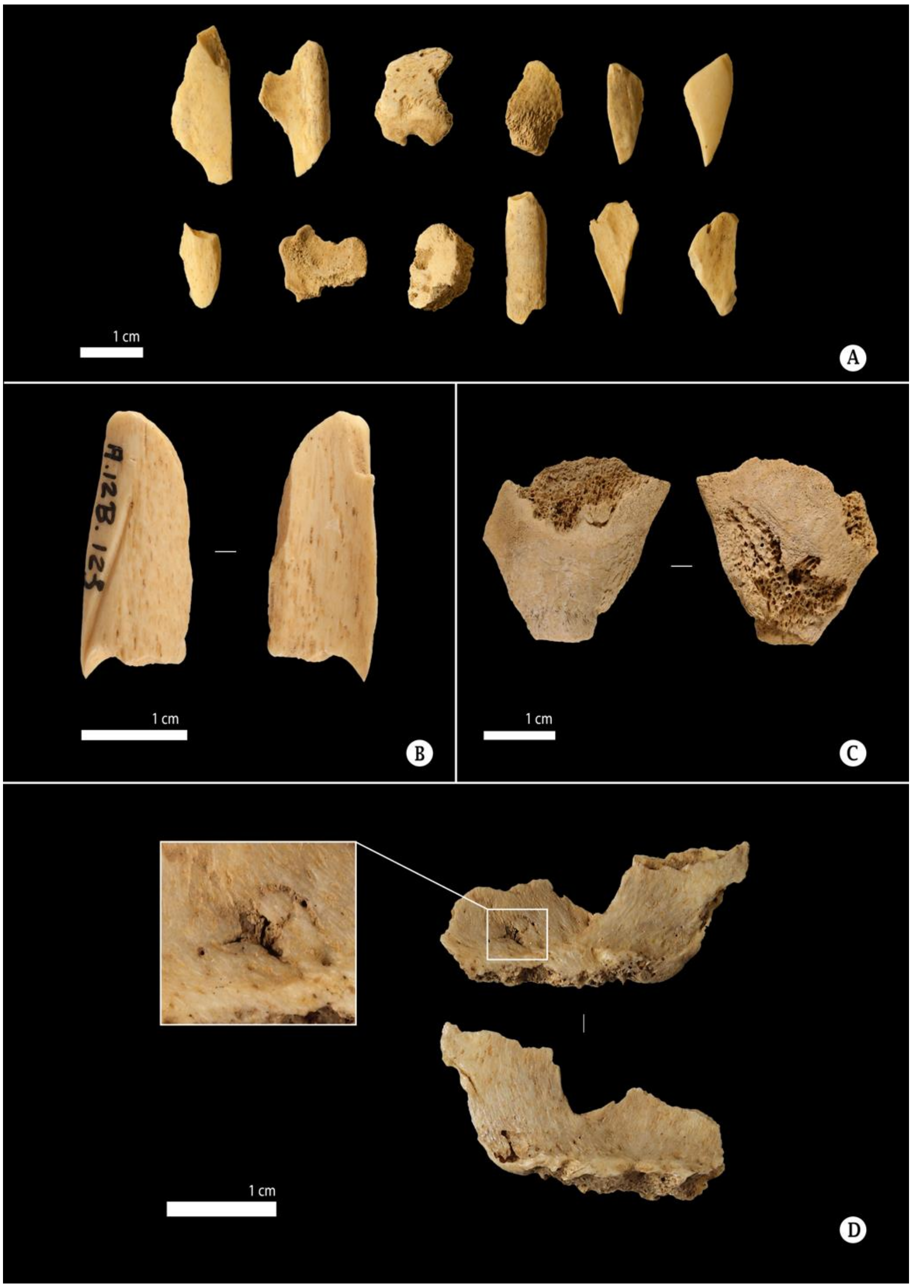

Figure S14: Carnivore modifications identified within the non-identifiable faunal assemblage of Level VII at Amalda I.

As proposed by Yravedra, the hypothesis that chamois were brought to the cave by a medium-sized felid such as lynx or leopard cannot be discarded, according to the available evidence such as the type of prey, the age and skeletal profiles and taphonomy of the bone assemblage, including also the cave location and ethology of the predator. However, a primary chamois accumulation by humans might also be considered, with scavenging by carnivores after humans left the carcasses. Among the diagenetic processes that affected the deposit, 12% of the non-identifiable fragments showed bone surface erosion effected by water dissolution and 6% of them had mineral dendrites of manganese, which might indicate periods of ponding during the burial process of the organic materials, while weathering is scarcely identified (only 1% of the set), indicating a rapid burial process. Finally, it is remarkable to have identified 4% of the bones as polished, likely related to the evidence of cave bear presence evidence at the site.

SUPPLEMENTARY INFORMATION – section 4

Database used for the spatial analysis performed at Amalda I site:

http://dx.doi.org/10.17632/vvyk8m5ttx.2

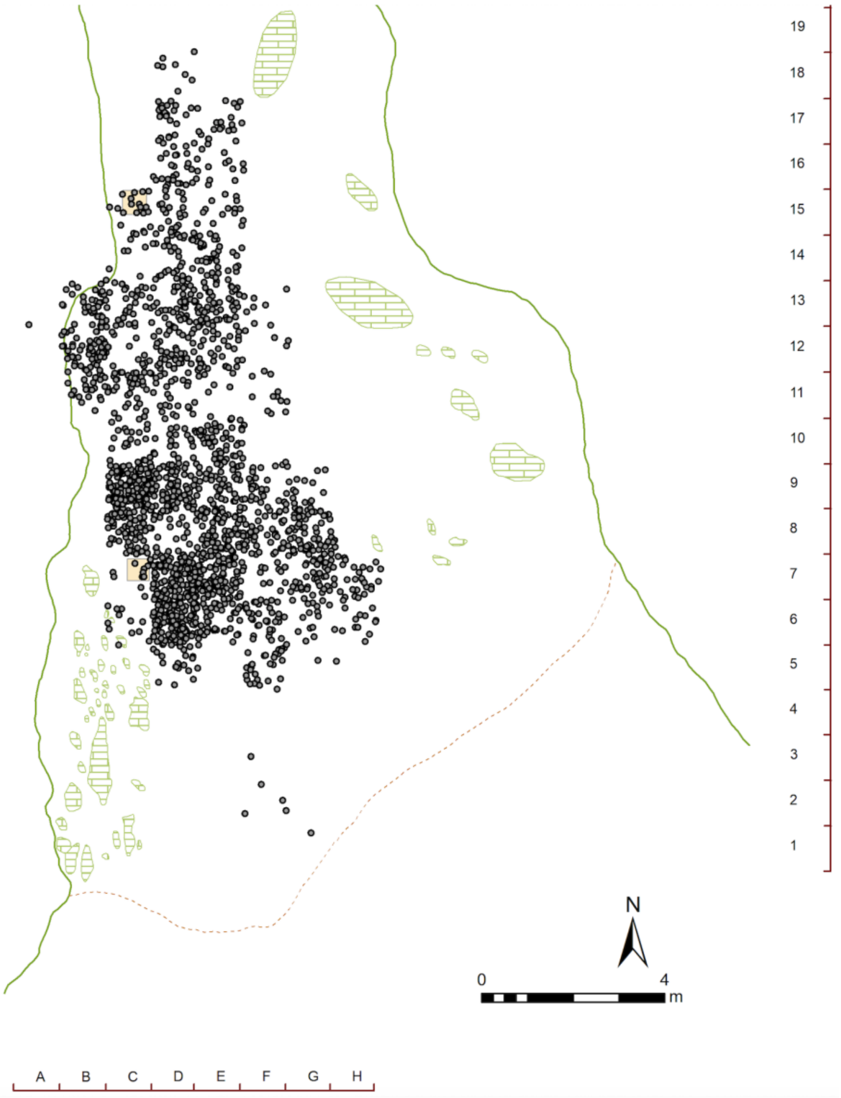

Figure S15: Distribution plan of the Amalda I assemblage.

References

1. Altuna, J. (Ed.). *La cueva de Amalda (Zestoa, País Vasco). Ocupaciones paleolíticas y postpaleolíticas* (Monografía. Colección Barandiarán 4, 1990).
2. Baldeón, A. Las industrias de los niveles paleolíticos. In *Ocupaciones paleolíticas y postpaleolíticas* (eds. Altuna, J.), 63-115 (Monografía. Colección Barandiarán 4, 1990).
3. Rios-Garaizar, J. *Industria lítica y sociedad del Paleolítico Medio al Superior en torno al Golfo de Bizkaia* (Publican. Universidad de Cantabria, Santander, 2012)
4. Areso, P., Aranzasti, M., Olaskoaga, M. & Uriz, A. (1990). Sedimentología de la cueva de Amalda. In *Ocupaciones paleolíticas y postpaleolíticas* (eds. Altuna, J.), 33-48 (Monografía. Colección Barandiarán 4, 1990).
5. Yravedra, J. Subsistencia en el Paleolítico Superior Inicial de la Cornisa Cantábrica. *Gallaecia*, 45-60 (2002).
6. Yravedra, J. Acumulaciones biológicas en yacimientos arqueológicos: Amalda VII y Esquilleu III-IV. *Trabajos de Prehistoria* **63(2)**, 55-78 (2006).
7. Yravedra, J. Nuevas contribuciones en el comportamiento cinegético de la Cueva de Amalda. *Munibe* **58**, 43-88 (2007).
8. Geiling, J.M., Marín-Arroyo, A.B., Straus, L.G. & González-Morales, M.R. Deciphering archaeological palimpsests with bone micro-fragments from the Lower Magdalenian of El Mirón cave (Cantabria, Spain). *Hist. Biol.* **30(6)**, 730-742, doi: 10.1080/08912963.2017.1385611 (2017).
9. Marín-Arroyo, A.B. *Arqueozoologia en la parte oriental de Cantabria durante la transición Pleistoceno /Holoceno: La Cueva de El Mirón* (Publican. Universidad de Cantabria, Santander, 2010).
10. De la Torre, I. & Wehr, K. Site formation processes of the early Acheulean assemblage at EF-HR (Olduvai Gorge, Tanzania). *J. Hum. Evol.* **120**, 298-328 (2018).
11. Rios-Garaizar, J. Organización económica de las sociedades neandertales: El caso del nivel VII de Amalda (Zestoa, Gipuzkoa). *Zephyrus* **LXV**, 15-37 (2010).
